# Podoplanin-defined tumour plasticity and CCR7-mediated lymphatic metastasis in triple-negative breast cancer

**DOI:** 10.1038/s41416-026-03402-4

**Published:** 2026-04-09

**Authors:** Zhi Wang, Lise Martine Ingebriktsen, Tove Bekkhus, Lyusong Ma, Antonio Queiro-Palou, Wenyang Shi, Anastasia Bazioti, Michail Angelos Panagias, Martina Vigorelli, Joakim Harry Lehrstrand, Sarah Ring, Anna Dimberg, Elisabeth Wik, Erling Andre Hoivik, Jonas Fuxe, Maria H. Ulvmar

**Affiliations:** 1 Department of Medical Biochemistry and Microbiology (IMBIM), Uppsala University, Uppsala, Sweden; 2 Department of Oncology-Pathology, Karolinska Institutet, Stockholm, Sweden; 3 Division of Pathology, Department of Laboratory Medicine, Karolinska Institutet, Huddinge, Sweden; 4 Division of Molecular Medicine, Department of Medical and Translation Biology, Umeå University, Umeå, Sweden; 5 Rudbeck Laboratory, Department of Immunology, Genetics and Pathology, Uppsala University, Uppsala, Sweden; 6 Science for Life Laboratory, Uppsala, Sweden; 7 Section for Pathology, Department of Clinical Medicine, Centre for Cancer Biomarkers CCBIO, University of Bergen, Bergen, Norway; 8 Department of Pathology, Haukeland University Hospital, Bergen, Norway

**Keywords:** Breast cancer, Metastasis, Tumour heterogeneity

## Abstract

**Background:**

Lymphatic metastasis is strongly associated with poor prognosis. Although the chemokine receptor CCR7 is a well-established promoter of lymphatic dissemination, its prognostic relevance remains weak. We show that tumour cell plasticity, defined by podoplanin (PDPN)-expression and promoted by hypoxia, intersects with CCR7 function in triple-negative breast cancer (TNBC).

**Methods:**

In vivo and in vitro studies using a CCR7-expressing TNBC mouse model were combined with transcriptomic profiling. Human relevance was assessed using scRNA-seq datasets from cell lines and primary tumours, as well as METABRIC breast cancer cohorts.

**Results:**

A PDPN-defined tumour cell mesenchymal shift, promoted by hypoxia, was required for efficient CCR7-driven lymphatic metastasis and tumour progression. PDPN-expression was linked to tumour cell collagen-expression and suppression of interferon-signalling, features associated with an immune-cold microenvironment. PDPN-expression with effects on interferon and collagen programmes was observed across murine and human TNBC cell lines and correlated with hypoxia signatures in primary TNBC, mirroring murine findings. In METABRIC, a high combined CCR7–PDPN score predicted poor survival in lymph node–positive patients, whereas either marker alone lacked prognostic value.

**Conclusions:**

PDPN is a tumour cell-associated biomarker of plasticity in TNBC, revealing synergy between hypoxia-induced mesenchymal phenotypic shifts and CCR7 in promoting lymphatic dissemination and poor prognosis.

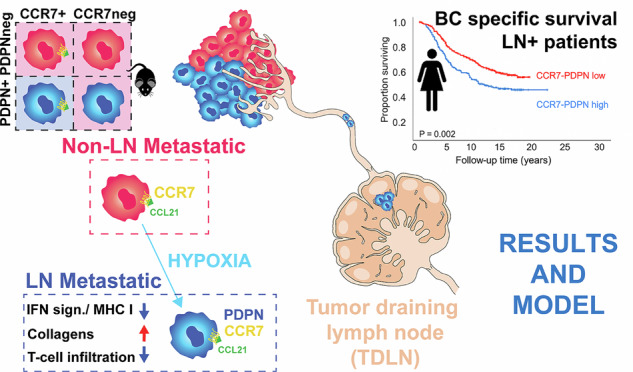

## Background

Lymph node (LN) metastasis serves as an independent prognostic indicator in several cancer types, including breast cancer, strongly associated with an increased risk of disease progression and distant metastases [1, 2]. Both intrinsic and extrinsic factors contribute to lymphatic metastasis, including epithelial-to-mesenchymal transition (EMT), immune evasion, metabolic reprogramming, and microenvironmental conditioning [3–6]. The factors that determine tumour spread and their interactions may vary across different tumour types and stages [7]. These factors can also influence subsequent processes, including the risk of distant metastatic spread and systemic tumour immunity.

Tumour cell expression of C-C chemokine receptor 7 (CCR7) has been identified as a tumour cell intrinsic factor that promotes lymphatic metastasis, as demonstrated both in human cancers and experimental work [8–12]. CCR7 enables tumour cells to exploit chemotactic signalling pathways normally used by immune cells to migrate into lymphatic vessels [13, 14] and subsequently transit into tumour-draining lymph nodes (TDLNs). Upregulation of CCR7 in mammary tumour cells, and its ligand C-C motif chemokine ligand 21 in lymphatic endothelial cells (LECs), has been linked to Transforming Growth Factor beta 1 (TGF-β1)-induced EMT and lymphatic metastasis [15]. Data from human breast cancer suggest that the effect of tumour-expressed CCR7 on driving lymphatic metastatic spread may be limited by tumour stage or subtype and microenvironmental factors [16, 17], but details are still lacking.

Here we provide new insights into the interplay between CCR7 in lymphatic metastasis promotion and tumour cell plasticity using EO771 (also known as E0771), a model of triple negative (TN)-like mammary carcinoma [18, 19]. Similar to many carcinoma models in the C57Bl6 background, EO771 display limited lymphatic metastasis [18–20]. Our data suggests that a phenotypic shift characterised by enhanced mesenchymal tumour-cell features, marked by upregulation of the glycoprotein podoplanin (PDPN), is required for efficient CCR7-driven metastasis. PDPN is a tumour stromal marker classically associated with fibroblasts and lymphatic vessels [21, 22]. Tumour cell expression of PDPN has been described in certain malignancies, such as melanoma and head and neck carcinoma, where it is often linked to invasive behaviour [21]. However, spontaneous tumour cell-specific PDPN upregulation has not, to our knowledge, previously been reported in breast cancer.

Importantly, EMT is not a binary switch, but rather a process that can display reversibility and adaptation to the microenvironment [23, 24]. Our data support this notion by coupling spontaneous PDPN-induction and gain of mesenchymal tumour traits in hypoxia. In line with that, epithelial-to-mesenchymal plasticity in tumour cells has been linked to adaptation to different growth conditions, metastasis, and immune evasion [23, 24]. Cell-specific changes included suppression of baseline interferon signalling. The latter is an effect that is further synergistically enhanced in cells co-expressing CCR7 and PDPN. We further show that PDPN expression is heterogeneous across mouse and human breast cancer cell lines and is present in human breast cancer, particularly TNBC. Key molecular effects linked to PDPN upregulation in the EO771 model are also recapitulated in human breast cancer, including modulation of collagen expression. The relevance of the CCR7-PDPN tumour axis to human breast cancer is also reinforced by biomarker analyses of mRNA CCR7–PDPN co-expression in METABRIC breast cancer patient cohorts.

Taken together, our findings support an impact of hypoxia and tumour cell plasticity in facilitating CCR7-driven tumour dissemination, suggesting CCR7-PDPN synergy in aggressive breast cancer progression and poor prognosis.

## Materials and methods

### Cell culture and cell sorting

CCR7-expressing EO771 and vector control, EO772 X2, were generated through retroviral transduction. PLNCX2 (Takara Bio USA, Inc., San Jose, CA, USA) vectors containing mouse *Ccr7* cDNA and the tdTomato reporter gene were transduced into EO771 mouse mammary carcinoma cells (CH3 BioSystems LLC, Amherst, NY, USA), and tested for mycoplasma. Retrovirus production and transduction were performed using Phoenix ampho-helper-free cells as described [25]. Cells expressing tdTomato and CCR7 were enriched by fluorescence-activated cell sorting (FACS) on a BD FACSAria III (BD Biosciences, San Jose, CA, USA). CCR7 was detected using anti-CCR7-APC (4B12), and dead cells were excluded using SYTOX™ Blue dead cell stain (Thermo Fisher Scientific, Waltham, MA, USA). Further sorting was conducted to isolate cell populations based on spontaneous upregulation of PDPN using anti-PDPN-eFluor™660 (eBio8.1.1, Thermo Fisher Scientific). No clonal selection was performed. Over the course of repeated experiments, both CCR7 expression and PDPN expression were controlled for by flow cytometry. Cells were maintained in RPMI-1640 with HEPES (Gibco™_,_ Thermo Fisher Scientific), supplemented with 10% foetal bovine serum, 1% L-glutamine, and 1% penicillin-streptomycin (all Gibco™) at 37 °C in a humidified incubator with 5% CO₂. 1 mg/ml G418 (Invitrogen, Thermo Fisher Scientific) was used for selection. Proliferation was measured by seeding a total number of 0.5 × 10^5^ cells per well in triplicates in a 12-well plate. Cells were harvested at 24, 48, and 72 h. Live cells were counted using Countess II Automated Cell Counter or CyQUANT® Cell Proliferation Assay Kit (Invitrogen, Thermo Fisher Scientific) according to the manufacturer’s instructions. Hypoxia in culture was enzymatically induced by using a combination of glucose oxidase (GOX) and catalase (CAT), following the established GOX/CAT method for induction of hypoxia [26]. Shortly, GOX (G6125-10KU, Sigma-Aldrich (Merck), St. Louis, MO, USA) was prepared as a 1:10,000 (w/v) dilution, while CAT (C30-100MG, 23,100 U/mg, Sigma) was diluted 1:5000 (v/v) in culture medium. Cells were treated for 8 h, before analysis of PDPN expression by flow cytometry.

### RNAseq and bioinformatic analysis of cell lines

RNA sequencing was conducted at the Bioinformatics and Expression Analysis core facility at the Karolinska Institute, Sweden. Total RNA was extracted from EO771 samples (4 technical replicates), using the RNeasy Mini Kit (Qiagen, Valencia, CA) following the manufacturer’s instructions. Libraries were generated using the TruSeq Stranded mRNA kit (Illumina) and sequenced on an Illumina NextSeq 550 platform in single-end mode, producing 75 bp reads mapped to the mouse reference genome (GRCm38/mm10).

Sequencing was run in two batches: the first batch included X2, CCR7, CCR7-PDPN, and the second batch included X2, X2-PDPN, and CCR7-PDPN. All the data was analysed using Python (v.3.11.5). Normalisation was performed using Transcripts Per Million by rnanorm (v.1.5.1). Subsequently, principal component analysis (PCA) was conducted using sklearn (v.1.3.0) to reduce dimensionality and visualise the data, particularly by observing batch effects and sample variations. Differentially expressed genes (DEGs) analysis was performed using pyDESeq2 (v.0.4.4). DEGs exhibiting an absolute log2 fold change >1 (|log2FC| > 1) and adjusted *p* value < 0.05 or FDR < 0.05 were regarded as significant. Gene set enrichment analysis (GSEA) was performed using the GSEApy (v.1.1.9), with mouse hallmark gene sets (v2023.2) obtained from the Molecular Signatures Database (MSigDB).

Single-cell RNAseq data from Wu et al. [27] were downloaded from CZ CELLxGENE at https://cellxgene.cziscience.com/collections/dea97145-f712-431c-a223-6b5f565f362a. The raw count from this study, GSE173634, was accessible via the National Centre for Biotechnology Information Gene Expression Omnibus database. PDPN-positive TNBC patient ID 4513 was analysed. The processed scRNA-seq of human breast cancer cell lines was available on figshare (https://figshare.com/articles/dataset/Single_Cell_Breast_Cancer_cell-line_Atlas/15022698?file=33943715). Data normalisation and dimensionality reduction, using default parameters, were conducted using scanpy (v.1.9.6). More specifically, dimensionality reduction was performed using Uniform Manifold Approximation and Projection (UMAP), and the clustering was conducted using the Leiden algorithm with a resolution of 0.1. The Wilcoxon rank test was applied to identify DEGs between clusters, and significant genes were identified with a Bonferroni–Hochberg corrected *P* value < 0.05 and |log2FC| > 1. Pathway analysis was performed using GSEApy (v.1.1.9) with human hallmark gene sets (v2023.2) obtained from the MSigDB.

### Mouse models and tumour inoculation

All animal procedures were approved by the Uppsala County Regional Ethics Committee (Dnr: 6009/17 and 5.8.18-03169/2023 MHU and 5.8.18-16487/2024 AD). Female C57BL/6J mice and *Prox1-EGFP* mice [29] housed under specific pathogen–free conditions with cage enrichment, age 8–20 weeks, were used in the experiments. Group sizes were determined from pilot data of 3–4 mice. 4 × 10^5^ cells in 5 μL PBS (Gibco) were injected into the 4th mammary fat pads. Tumour-bearing mice were sacrificed at a defined experimental endpoint or when tumours reached max 12 mm in diameter, measured by calliper, in accordance with recommended guidelines [30]. Animals were monitored daily, and inclusion/exclusion criteria were defined a priori according to the university’s veterinary scoring system for monitoring animal health and human endpoints. No formal randomisation or blinding procedure was used. Nude (*AthymicNude-Foxn1nu*) mice received subcutaneous injection of 1 million cells, and tumour growth was followed up to max ethical end point 1.5 cm^3^. To assess hypoxia, the Hypoxyprobe-PAb27 Kit (Hypoxyprobe Inc., Burlington, MA, USA) was used following the manufacturer’s instructions. Shortly, mice were intraperitoneally (i.p.) injected with pimonidazole hydrochloride at a dose of 60 mg/kg body weight, prepared in sterile PBS. The injection was administered 60 min before euthanasia. Tissues were fixed in 0.8% PFA overnight and processed by cryo-sectioning. Hypoxia was detected using the PAb27 monoclonal antibody from the kit, followed by immunofluorescence staining.

### Flow cytometry

Tumour digestion was performed as previously described [31]. Data were acquired on a BD FACSAriaIII flow cytometer (BD Biosciences) or Cytoflex (Beckman Coulter Inc., Brea, CA, USA) and analysed using FlowJo software (FlowJo, LLC, version 10.6.1). Antibodies are listed in Supplementary Table 2.

### Tissue preparation and imaging

Mouse tissue sections for imaging were prepared by cryosectioning and stained as described [31, 32]. Alternatively, after fixation, tissues were embedded in agarose and vibratome sections, 80 μm were prepared. In addition, formalin-fixed and paraffin-embedded biobank LNs from breast cancer patients were stained as described [28, 32]. Antibodies are listed in Supplementary Table 2. Nuclear staining was performed using 4′,6-diamidino-2-phenylindole (DAPI, Invitrogen). Slides were mounted using Prolong™ Gold Antifade Mounting Media (Thermo Fisher Scientific). Images were acquired using an automated slide scanner, PhenoImager (Vectra Polaris) (Akoya Biosciences, Marlborough, MA, USA). Images for publishing were prepared using the open-source Fiji^TM^ software (ImageJ v.2.11.0).

### Image analysis

Immune cell analysis: Tumour sections (central region) were analysed in Fiji (ImageJ 2.11.0). Random 0.21 mm² ROIs were selected, excluding margins and necrosis. CD3⁺CD8⁺ cells were manually counted, while CD11b⁺ and MHC-II⁺ cell densities were quantified using a standardised macro.

### METABRIC gene expression analyses

We analysed a gene expression dataset from primary breast cancer with clinico-pathological data and follow-up information: Molecular Taxonomy of Breast Cancer International Consortium (METABRIC), METABRIC discovery (*n* = 939) and validation cohort (*n* = 845) [33]. Intrinsic molecular subtypes based on the PAM50 classification were available [34]. The normal-like category was excluded. JExpress/2012 (www.molmine.com) was used to extract DEGs from the METABRIC cohort. In cases of multiple probes per gene symbol in the gene expression matrices, the probes were collapsed according to maximum probe expression per gene [35].

### Statistical methods METABRIC

SPSS (version 25.0, IBM Corp., Armonk, NY, USA) was used. Spearman’s rank correlation test was applied to compare bivariate continuous variables, and Spearman’s correlation coefficients (*ρ*) were reported. Mann–Whitney *U* or Kruskal–Wallis tests were applied to analyse the differences in the distribution of continuous variables between two or more categories. For univariate survival analyses, including death from breast cancer or recurrence from breast cancer as endpoints, the Kaplan–Meier product-limit method (log-rank test for differences) was applied. In multivariate logistic regression analysis, the calculations were done according to the Backward Elimination (Likelihood Ratio), with *p* values derived from Step 1 in the ‘model if term removed’-table. Moreover, only patients with information on all the variables were included. All statistical tests were two-sided, and statistical significance was assessed at 5% level. CCR7-PDPN score dichotomisation; quartiles 1–3 = low CCR7-PDPN score; quartile 4 = high CCR7-PDPN score.

## Results

### Expression of CCR7 in the triple-negative breast cancer cell line EO771 leads to increased tumour growth and lymphatic metastasis in vivo

**Fig. 1:**
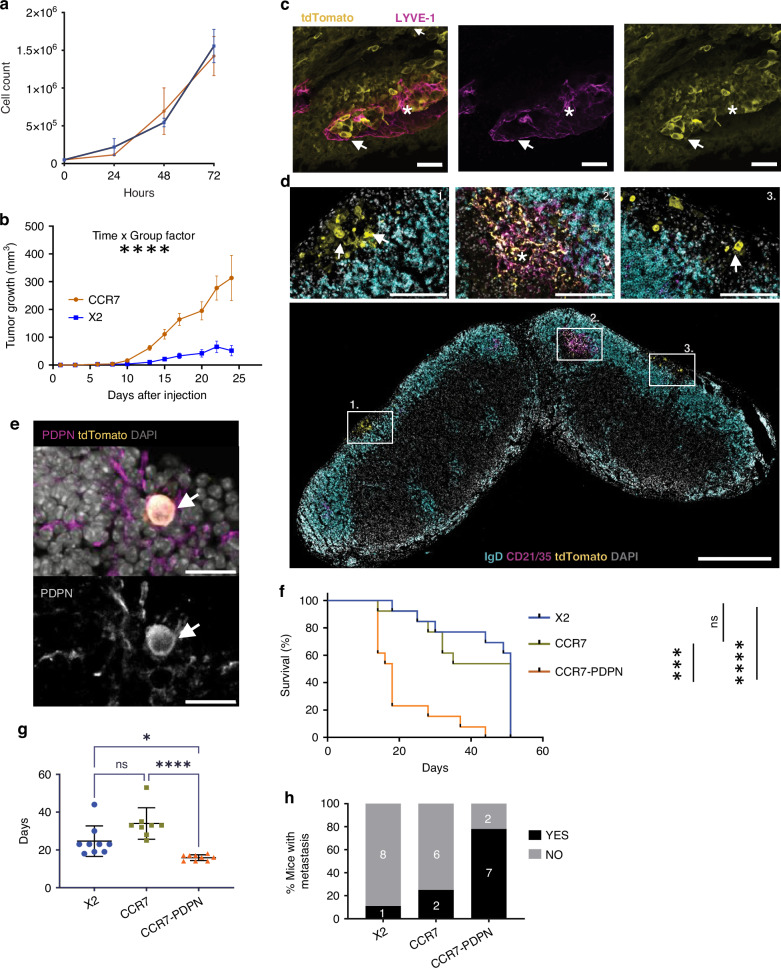
Spontaneous upregulation of PDPN is required for efficient lymphatic metastasis and increased growth of CCR7-expressing EO771. **a** In vitro proliferation of EO771 X2 control (blue) and CCR7-expressing cells (orange). Data represent mean ± SD of three replicates; no significant differences were observed (two-way RM ANOVA ns). Representative of two experiments. **b** In vivo growth after mammary fat pad injection (*n* = 15/group). Mixed-effects model (REML, Geisser–Greenhouse correction) showed significant time × group interaction (*P* < 0.0001) and post hoc differences from day 10 onward. **c** Vibratome section of CCR7 tumour stained for LYVE-1 (magenta) and tumour cells (tdTomato, yellow). Tumour cells (arrow) are observed within lymphatic vessels (star indicates lumen). Representative of 3 tumours. Scale bar: 50 µm. **d** tdTomato+ metastatic cells in TDLNs from CCR7 tumours (insets 1, 3), and tdTomato+ immune complexes on FDC networks (CD21/35) in germinal centres (inset 2). IgD marks B cell zones. Scale bars: overview 500 µm, insets 100 µm. Representative of 9 LNs. **e** Confocal image showing PDPN (magenta) on CCR7-expressing tdTomato+ tumour cells in TDLNs. Scale bar: 20 µm. Representative of 3 analysed TDLNs. **f** Survival analysis after fat pad injection. Tumours were allowed to grow to 9 mm in the largest diameter. Log-rank Mantel-Cox test showed reduced survival in CCR7-PDPN vs. X2 (****p < 0.0001) and CCR7-PDPN vs. CCR7 (***p = 0.0003). *n* = 13 per group. **g** Timepoints of tumour sampling for metastatic assessment in (**h**) differed significantly (Kruskal–Wallis, *p < 0.05; ****p < 0.0001). **h** Metastasis in TDLNs was quantified by manual assessment of cryosections (≥3 levels/LN) from size-matched tumours (i.e. 262 ± 119 mg): X2 (*n* = 9), CCR7 (*n* = 8), CCR7-PDPN (*n* = 9); 62–100 sections per group.

CCR7 expression is known to increase lymphatic metastasis across multiple tumour models and has been associated with lymphatic dissemination in several types of human cancers [36]. To condition the mammary carcinoma cell line EO771 for enhanced metastatic potential, expression of the chemokine receptor CCR7 was introduced (Supplementary Fig. 1A) together with tdTomato for sensitive tracing of tumour cells. CCR7 expression in EO771 did not affect cell growth under normal culture conditions in vitro (Fig. 1a) but EO771-CCR7 injected into the mammary fat pad displayed a dramatic increase in tumour growth in vivo compared to the control (Fig. 1b). In primary tumours, lymphatic vessels were restricted to the tumour margins, where an association between tdTomato positive tumour cells and lymphatic vessels was observed (Fig. 1c). Two main tdTomato sources were observed in the TDLNs: metastatic tumour cells in the subcapsular sinus (SCS), sometimes extending into the LN parenchyma (Fig. 1d insets 1 and 3); and tdTomato-containing immune complexes on Follicular Dendritic Cells (FDCs), i.e. CD21/35+ cells, in B-cell follicles (Fig. 1d inset 2), previously linked to B-cell–driven tumour progression in this model [37]. tdTomato was also detected in SCS macrophages (Supplementary Fig. 1B).

### Spontaneous upregulation of PDPN is required for efficient lymphatic metastasis and growth of CCR7-expressing EO771

**Fig. 2:**
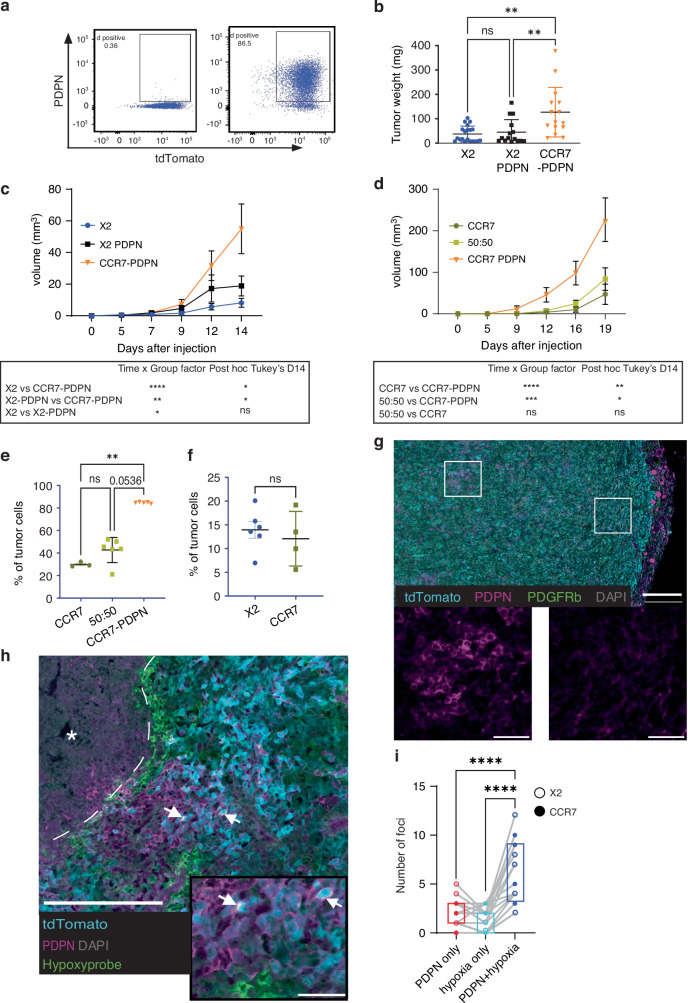
PDPN upregulation in EO771 does not drive tumour growth without CCR7 and is associated with the hypoxic microenvironment. **a** Original proportion of PDPN-positive cells in EO771 vector control X2 0.36% (left) and an increased proportion established by FACS sorting 86.5% (right). **b**, **c** Mice injected bilaterally with EO771-X2 (*n* = 12), X2-PDPN (*n* = 8), or CCR7-PDPN (*n* = 8) cells in the 4th mammary fat pad; tumour growth followed to day 14. **b** Tumour weight at end stage (day 14) for the different groups. *n* = 20 X2, *n* = 15 X2-PDPN, *n* = 15 CCR7-PDPN. Kruskal–Wallis test and Dunn multiple comparison. **p < 0.01. **c** Growth curves analysed by two-way RM ANOVA and mixed-effects model (REML, Geisser–Greenhouse correction). CCR7-PDPN tumours grew faster than X2 (*P* < 0.0001) and X2-PDPN (*P* < 0.01); post hoc differences at day 14 were displayed. **d** Tumour growth of CCR7, CCR7-PDPN, and 1:1 CCR7/CCR7-PDPN mixtures (*n* = 5 mice/group, bilateral). Two-way RM ANOVA showed significant group and time × group effects for CCR7-PDPN (*P* < 0.0001); no difference between 50:50 and CCR7-PDPN; post hoc differences at day 14 were displayed. **e** PDPN proportion in end-stage tumours. Kruskal–Wallis test and Dunn multiple comparison were used for statistical analysis. **p < 0.01. **f** Induction of PDPN analysed by FACS in size-matched tumours from mice injected with PDPN-negative X2 vector control or CCR7-positive EO771. No significant differences. **g** Staining of EO771 X2 tumour for tdTomato (cyan), PDPN (Magenta), PDGFRbeta (green) and Nuclei (DAPI grey). Scale bar 200 μm. Insets show central foci (1) with PDPN expression and peripheral foci (2) with low or no detection. Scale bar 50 μm. **h** Staining of EO771-CCR7 tumour for hypoxy-probe (green) together with detection of tdTomato (cyan), PDPN (magenta), Nuclei (DAPI grey). Dotted line mark border between healthy and necrotic (star) tissue. Hypoxy-probe staining lines the necrotic areas. PDPN is seen expressed in cells adjacent to these areas, and in rare hypoxic cells (arrows). Scale bar 200 μm, inset 50 μm. **i** Paired analysis (one-way ANOVA) shows an association of PDPN and hypoxia. *n* = 17 tumour sections from *n* = 4 tumours. Closed circles: CCR7-expressing tumours and open circles vector control (X2). ****p = <0.0001.

Staining TDLNs for the glycoprotein podoplanin (PDPN), a marker of LN fibroblastic reticular cells and lymphatic vessels [21, 22], serendipitously revealed PDPN expression in metastatic tumour cells (Fig. 1e). Analysis by flow cytometry demonstrated a subpopulation of tumour cells with upregulation of PDPN in CCR7-expressing EO771, and scattered cells in the vector control (X2) (Supplementary Fig. 2A). To explore a possible contribution of the PDPN positive phenotype to LN metastasis driven by CCR7, we sorted PDPN positive and negative cells from CCR7-expressing EO771 (Fig. 2a). PDPN-expression remained stable over time in cell culture. tdTomato expression levels were controlled for across the cell derivatives, as were CCR7 levels (Supplementary Fig. 2B, C).

Strikingly, survival based on tumour size was dramatically reduced in mice injected with CCR7-PDPN EO771 (18 days) compared to CCR7 or X2 (both 51 days) (Fig. 1f). Tumour-take rates were also higher in CCR7-PDPN (92.3%) compared to CCR7 (61.5%) and X2 (38.4%) EO771. The latter was consistent across multiple experiments, with an average 50% for X2, 70% for CCR7 and 80% in CCR7-PDPN (Supplementary Fig. 2D). In contrast, no differences in tumour cell proliferation were observed in cell culture (Supplementary Fig. 2E).

To rule out the possibility that larger tumour size alone influenced metastasis, we compared metastasis from mice with primary tumours selected for a medium size range (i.e 262 ± 119 mg across groups), and by sectioning and imaging several levels of each TDLN. Imaging allows differentiation of tdTomato-positive metastatic cells from tdTomato-positive immunocomplexes on FDCs (Fig. 1d inset 2) and macrophages (Supplementary Fig. 1B). Despite a significantly shorter tumour growth period (Fig. 1g), CCR7–PDPN tumours showed higher metastatic spread to TDLNs than CCR7 or X2 controls (Fig. 1h). In contrast, tdTomato accumulation on FDCs, LN size, and germinal centre formation in the same TDLNs did not differ between groups (Supplementary Fig. 3A–E). The latter was also the case when tumours were harvested at the same time point (day 14) (Supplementary Fig. 3F–I). The accumulation of tdTomato across the GC or FDC area (an indicator of ongoing GC formation and B-cell activation [38]) was similar across groups (Supplementary Fig. 3J). Hence, GC formation is independent of tumour size, stage and metastatic spread to the TDLNs. Interestingly, in the rare cases of metastasis from PDPN-negative CCR7-positive tumours, the resulting micro-metastases consisted of PDPN-positive tumour cells, suggesting in vivo selection or induction of PDPN expression. (Supplementary Fig. 4).

### Spontaneous PDPN upregulation occurs in vivo in EO771 but requires CCR7 to drive metastasis and growth

To test whether PDPN upregulation alone can drive metastasis in EO771, PDPN-positive vector control cells (X2-PDPN) were stably enriched by cell-sorting (~80–90%; Supplementary Fig. 2A, Fig. 2a). PDPN-expression increased tumour take from 65.7% to 83% at day 14 (Supplementary Fig. 5A), but in contrast to CCR7-PDPN, X2-PDPN did not display increased tumour growth (Fig. 2b, c, Supplementary Fig. 5B), and did not form LN metastases (Supplementary Fig. 5C).

To test whether CCR7-PDPN cells have a growth advantage in vivo, a 50:50 mix of CCR7 and CCR7-PDPN cells was injected, with single-population controls. No selection for CCR7-PDPN was observed, indicating no direct growth advantage (Fig. 2d, e). Notably, PDPN-positive cells also appeared in tumours derived from the injection of PDPN-negative CCR7 EO771 (Fig. 2e). The spontaneous induction of PDPN in vivo occurs equally in the X2 vector control and CCR7-expressing tumours (Fig. 2f), with individual tumours showing larger variation than between the groups (Fig. 2e, f).

### Spontaneous upregulation of PDPN occurs in tumour cell areas associated with hypoxia

In situ analysis of tumours from PDPN-negative cell lines showed PDPN-positive cells mainly clustered in the tumour core. (Fig. 2g). PDPN has been previously suggested to be linked to mammary stem cell functions [39]. However, we did not detect any difference in colony-forming assays in vitro (Supplementary Fig. 6). Intra-tumoral PDPN induction suggested a link to hypoxia. Hypoxyprobe injection confirmed hypoxic regions at the necrotic–viable tumour border (Fig. 2h; Supplementary Fig. 7). Hypoxyprobe-positive cells were detected along the border between live and necrotic tumour areas (Fig. 2h and Supplementary Fig. 7A). Quantification further supported a connection between hypoxia and in situ PDPN-expression (Fig. 2i), mainly confined to cells adjacent to hypoxic areas rather than hypoxic cells, consistent with recently described and predicted EMT shift in post-hypoxic tumour cells [40]. To explore a possible direct impact of hypoxia on the selection or induction of PDPN-positive cells, we used the established enzymatic glucose oxidase (GOX) and catalase (CAT) system (GOX/CAT) [26]. This is based on enzymatic consumption of oxygen with concomitant neutralisation of formed H_2_O_2_ [26]. GOX/CAT-induced hypoxia induced a small but reproducible and detectable upregulation of PDPN expression (Supplementary Fig. 7B, C). As also suggested in other models [40], while hypoxia may initiate a mesenchymal shift, other factors in the TME are also likely to contribute.

### CCR7- and PDPN-expression impact the EMT signature, collagen expression, and the homeostatic interferon response

PDPN could either be upregulated alone or be part of a differentiation programme with other molecular changes in EO771. To distinguish between these possibilities and further understand the molecular consequences of CCR7 expression and spontaneous PDPN upregulation in EO771, we performed comprehensive RNA profiling.

**Fig. 3:**
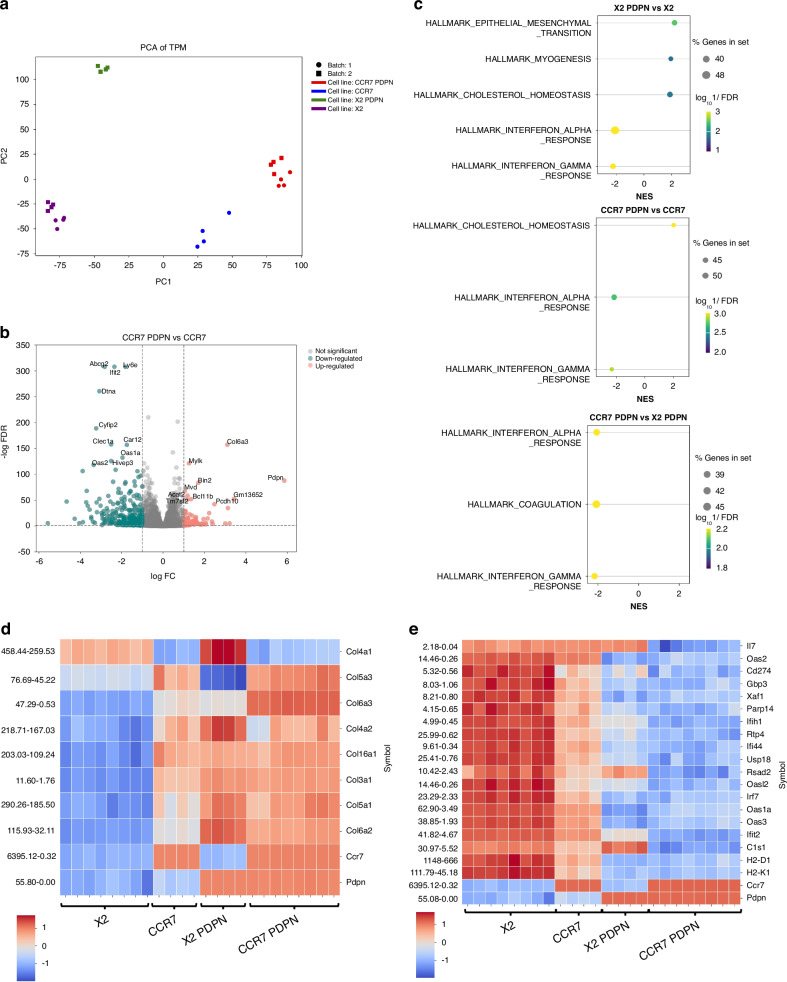
CCR7- and PDPN-expression impacts the EMT signature, collagen expression and the homeostatic interferon response. **a** PCA shows the distribution of four EO771 cell lines across two experimental batches. Data points are coloured and grouped by cell type and batch. **b** Volcano plots displaying DEGs between CCR7-positive and CCR7-PDPN-positive EO771 cells. Dashed lines indicate thresholds for upregulation and downregulation (∣Log2FC∣ > 1) and statistical significance (adjusted *p* value < 0.05). **c** Gene set enrichment analysis (GSEA) using mouse hallmark gene sets highlights differences between PDPN-positive and PDPN-negative cell lines. **d**, **e** Heatmaps displaying *Z*-scores of normalised gene expression, where *Z*-score = 0 represents the mean gene expression. Gene names are indicated on the right side, and normalised gene expression values are added on the left side of the heat maps for reference. **d** EMT-related collagens in CCR7 and PDPN-positive EO771 cell derivatives. **e** IFN-related and immune-related genes (i.e. *Il-7*).

PCA revealed distinct clustering of the four cell derivatives with and without CCR7 and PDPN (Fig. 3a). Differential expression analysis further identified sets of genes significantly up- or down-regulated between CCR7-PDPN and CCR7 cells (Fig. 3b), including several associated with immune modulation and extracellular matrix interactions. The tumour classification of EO771 mammary carcinoma has been debated, but it has most often been assigned as a TNBC or basal cell-like [18, 19] and display high degree of EMT and mesenchymal features [41]. Our analysis confirmed near-background ERα (*Esr1*) expression and no ERβ (*Esr2*), with very low levels of PR components, HER2 (*Erbb2*) and HER3 (*Erbb3*) (Supplementary Fig. 8). In contrast, EMT markers (*Vim, Twist1, Zeb1/2, Wnt7b*) were robustly expressed, while most cytokeratins were absent except Keratin 8 (*Krt8*). Notably*, ERα* and *Erbb2* expression were further reduced in CCR7-PDPN double-positive cells (Supplementary Fig. 8).

GSEA using mouse hallmark gene sets showed an enhanced EMT and myogenesis signatures in the X2-PDPN compared to the original X2 control (Fig. 3c). Rather than classical EMT markers, the top significantly upregulated DEGs in EO771 with PDPN expression encoded for collagens (Fig. 3b–d and Supplementary Table 1). The gene expression profile of collagens was further modulated by the co-expression of PDPN and CCR7, as illustrated by the changes of selected EMT-associated collagens (Fig. 3d). This pattern suggested further mesenchymal reinforcement or shift, in which PDPN is upregulated as part of differentiation toward a more mesenchymal tumour cell phenotype.

Among the top-ranked hallmark pathways (false discovery rate (FDR) < 0.05), only cholesterol homeostasis showed an increase in CCR7-PDPN EO771 (Fig. 3c). The top-ranked downregulated hallmark pathways included type I and type II Interferon (IFN) responses (Fig. 3c). Downregulation of IFN response-associated genes was evident in cells expressing either PDPN or CCR7 alone compared to the vector control and was further reduced in cells co-expressing both markers (Fig. 3c, e). CCR7-PDPN cells also showed a downregulation of *Il7* expression (Fig. 3e). The observed immune-modulation extended to Major Histocompatibility Complex (MHC) class I molecules, with reduced expression of H2-D1 and H2-K1 in CCR7-PDPN EO771. An overview of the top 100 up-regulated and down-regulated DEGs across the four cell lines is provided in Supplementary Table 1.

### CCR7 and PDPN double-positive tumours display a shift to a more immune-cold profile

**Fig. 4:**
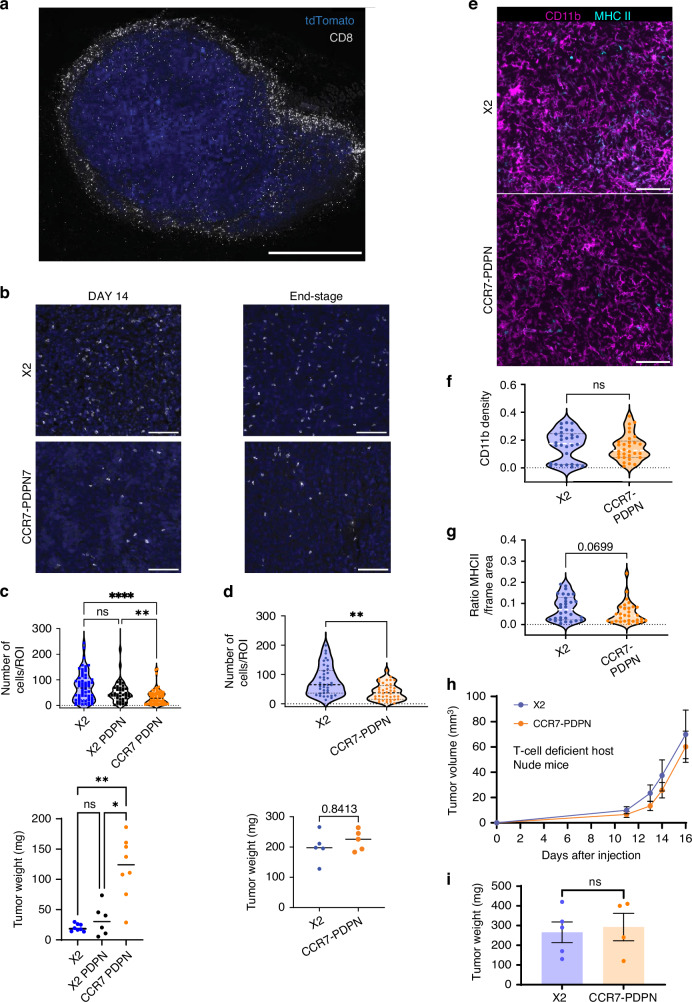
CCR7 and PDPN double-positive tumours display a shift to a more immune-cold profile based on reduced CD8 T-cell infiltration. **a** Overview of a tumour at an early stage, stained for CD8 (white) and tdTomato shown in blue. The tumour border has a high density of T-cells, while very few T-cells are found inside the tumour mass. Scale bar 1 mm. **b** Representative images of CD8 staining (white) and tdTomato in blue from central tumour areas. Day 14 and end-stage X2 and CCR7-PDPN tumours. Scale bar 100 μm. **c** Upper graph: Quantification of T-cell density on day 14 after tumour injection, 63, 48 and 72 (Regions of Interest ROIs) per group, *n* = 6–8 each group. Lower graph: Tumour weight of analysed samples *n* = 6–8. **d** Upper graph: quantification of T-cell density in end-stage size-matched tumours, 38 and 40 ROIs per group. *n* = 5 each group. Lower graph: tumour weight of analysed samples *n* = 8. Tumour weight of analysed samples, *n* = 5. **e** Representative images of CD11b (magenta) and MHC class II (cyan) in tumours from X2 vector control and CCR7-PDPN. Scale bar 100 μm. **f** Total density of CD11b in X2 vector control and CCR7-PDPN. *n* = 3 and 10 ROIs per tumour, total 30. **g** Total density of MHC class II positive cells. *n* = 3 each group and 10 areas per tumour, total 30. **a**, **b**, **f**, **g** Analysis includes random areas excluding the tumour border and necrotic areas. **h** Subcutaneous injection of X2 and CCR7–PDPN EO771 cells into T-cell-deficient athymic nude mice eliminates the differences in tumour growth. *n* = 5 per group. Tumour growth showed up to day 16. Two-way RM ANOVA. Non-significant. Representative of two experiments. **i** Tumour weight of tumours harvested from nude mice, *n* = 5 each group. No significant differences. Unpaired *t*-test Mann–Whitney, and Krusal–Wallis test. ****p < 0.0001, ***p < 0.001, **p < 0.01.

CCR7-PDPN cells were not selected over CCR7 cells in competitive experiments (Fig. 2d, e), suggesting that tumour progression is promoted by indirect mechanisms. Reduced spontaneous IFN signalling (Fig. 3c, e) led us to examine effects on the immune microenvironment. Tumour-infiltrating lymphocytes (TILs) distinguish immune-cold from immune-hot tumours, the latter generally associated with better prognosis and response to immunotherapy [42]. EO771 tumours formed a capsule rich in fibroblasts, myeloid cells and T cells, but with sparse TILs (Fig. 4a). Imaging analysis showed reduced intra-tumoral CD8⁺ TILs in CCR7-PDPN tumours compared to controls (Fig. 4c–f), both at early tumour stages shown across EO771 X2, X2-PDPN and CCR7-PDPN (Fig. 4c), and end-stage tumours X2 and CCR7-PDPN (Fig. 4d), consistent with an immune-cold phenotype. In contrast, CD11b⁺ myeloid cell density was unchanged (Fig. 4g). EO771 is generally considered an immunogenic cell line [43]. The selective reduction of CD8⁺ T-cell infiltration in CCR7–PDPN tumours, therefore, likely represents an active evasion mechanism. To directly test whether differences in tumour establishment and growth between control EO771 and CCR7–PDPN tumours were mediated by T cells, we used athymic *Nude* mice. In these T-cell-deficient hosts, CCR7–PDPN tumours no longer displayed reduced tumour establishment or increased growth kinetics compared to control X2 (Fig. 4h). These data together support a mechanism in which CCR7–PDPN tumours in an immune-competent host reshape the adaptive immune microenvironment, reducing T-cell infiltration.

Because T cells are a source of IFNs that can restrict lymphangiogenesis [44], reduced T-cell infiltration may indirectly enhance lymphangiogenesis. Flow cytometry of EO771 tumours in *Prox1-EGFP* reporter mice, however, detected only scattered EGFP LECs in both groups, though CCR7-PDPN tumours showed a modest increase compared to controls (Supplementary Fig. 9A–D). Total endothelial cell proportions were unchanged.

### Heterogeneous PDPN-expression in TNBC lines and association to EMT and hypoxia in human primary tumour scRNA-seq

**Fig. 5:**
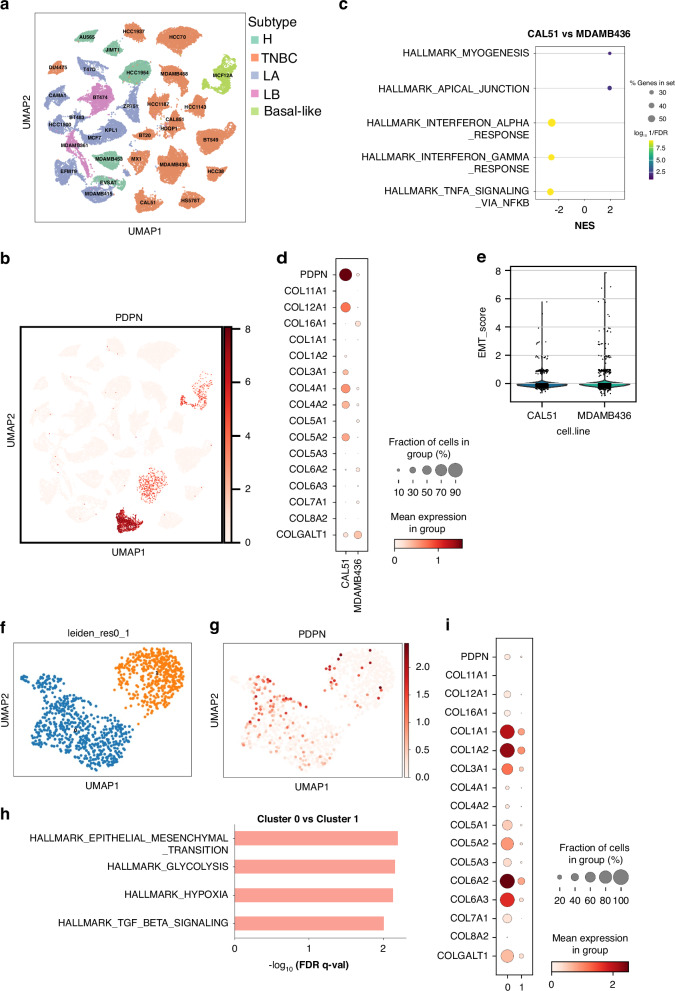
Human TNBC and basal-like cell lines and primary TNBC display heterogeneous PDPN expression linked to changes in collagen expression and hypoxia in vivo. **a** UMAP plot illustrating clusters from human breast cancer cell lines, coloured by subtypes, including TNBC, LA (luminal A), LB (luminal B), and basal-like (non-malignant cell line). **b** UMAP plot showing the PDPN expression among CAL-51 and MDA-MB-436. **c** GSEA using hallmark gene sets comparing CAL-51 (PDPN-high) with MDA-MB-436 (PDPN-low) TNBC cell lines, showing the top-ranked enriched up- and down-regulated pathways. **d** Dot plot showing the expression of PDPN and selected collagen genes in CAL-51 and MDA-MB-436. Dot size indicates the fraction of cells expressing the gene within each group, and colour intensity represents the mean expression level. **e** EMT score in CAL-51 and MDA-MB-436. **f** UMAP visualisation of single-cell transcriptomic data of a case of human primary metaplastic TNBC, based on Leiden clustering (resolution = 0.1) (Cluster 0, blue; Cluster 1, orange). **g** UMAP plot showing the expression of PDPN across the dataset, with higher expression levels concentrated in Cluster 1. **h** Hallmark pathway enrichment analysis comparing Cluster 0 and Cluster 1. **i** Dot plot showing the expression of PDPN and selected collagen genes in the two clusters. Dot size indicates the fraction of cells expressing the gene within each group, and colour intensity represents the mean expression level.

Although CCR7 has been extensively studied as a factor that promotes lymphatic metastatic behaviour in human breast cancer [8, 9, 11, 12], PDPN has not been thoroughly studied. To test whether our findings extend beyond EO771, we examined two additional mammary carcinoma cell lines: AT3 (C57Bl/6) and 4T1 (Balb/c). Both showed heterogeneous PDPN expression: AT3 had a large PDPN-positive population, and 4T1 had low but variable expression (Supplementary Fig. 10A). In both lines, CCR7 expression was mainly confined to PDPN-high cells. Next, we performed in-silico analyses to explore the potential translational relevance of PDPN expression in human breast cancer. First, a single-cell RNA sequencing (scRNA-seq) data set of 32 human breast cancer cell lines (Fig. 5a) [45]. Although several of the cell lines in this data set have been reported to express CCR7, i.e. MCF-7, DU-4475 and MDA-MB-361 [8, 12], CCR7 was not detectable at the single-cell mRNA level (data not shown and ref. [45]). PDPN was, however, detected in two TNBC cell lines, CAL-51 and MDA-MB-436, and in the basal-like pre-malignant cell line MCF-12A (Fig. 5b), the latter consistent with the known expression of PDPN in mouse mammary basal epithelial cells [39]. Both CAL-51 and MDA-MB-436 were originally isolated from metastatic sites [45] and are based on clustering transcriptionally related. Sub-clustering did not separate PDPN-positive from PDPN-negative cells (Supplementary Fig. 10B). GSEA using human hallmark gene sets comparing CAL-51 with MDA-BM-436 revealed reduced IFN signalling and upregulation of myogenesis hallmark in the PDPN enriched CAL-51, mimicking the findings in EO771 (Fig. 5c). Further, like in murine EO771 selected for PDPN-expression, CAL-51 cells display increase in EMT-associated collagens (Fig. 5d), while the overall EMT-score was similar between the both cell lines (Fig. 5e) in line with that PDPN expression reflects a mesenchymal shift rather than change in overall EMT. Extending the analysis to additional PDPN-negative, transcriptionally proximate clusters of human TNBC cell lines confirmed a consistent pattern: PDPN expression correlates with collagen upregulation and downregulation of IFN pathway genes, including reduced MHC class I expression compared to cell lines with low or no detectable PDPN (Supplementary Fig. 10C, D).

We next used a scRNA-seq data set of neoadjuvant-treated ER+, HER2+ and TNBC from 26 patients [27]. CCR7 was not detectable except in immune cells. However, PDPN-expression in tumour cells was confirmed in one patient with a metaplastic subtype of TNBC (Fig. 5g). To minimise inter-patient and treatment-related variability, we performed intra-patient analysis. Unsupervised Leiden gave the separation of the cancer cells into two clusters. PDPN cells were scattered in both clusters but were significantly enriched in cluster 0 (Fig. 5f, g). GSEA analysis using human hallmark gene sets showed upregulated hallmarks for Transforming Growth Factor beta (TGF-β), EMT and hypoxia (Fig. 5h). Collagen analysis revealed a distinct pattern of upregulation (Fig. 5i). Our data support that human TNBC breast cancer cell lines, as well as primary, can display heterogeneous upregulation of PDPN, which mirrors the effects we described in PDPN-positive mammary carcinoma EO771, including changes in collagen expression and shifts in the gene-expression profile of collagens.

While CCR7 has been observed to be expressed by breast cancer cells on the protein level in multiple studies [8, 9, 11, 12], PDPN-expression is a new finding. To further explore PDPN on the protein level, a set of 10 metastatic LNs from patients with invasive ductal carcinoma and TNBC [28, 32] was stained. Rare PDPN detection was found in clusters of cells associated with lymphatic vessels only in samples that displayed heterogeneous expression of cytokeratin (Supplementary Fig. 11A–C).

### In silico analyses show that high CCR7-PDPN mRNA expression score associates with aggressive tumour characteristics and presents independent prognostic value

Given synergistic effects between CCR7 and PDPN in the EO771 model for both tumour growth and LN metastasis, we further evaluated their cooperation in human breast cancer progression. Using the Molecular Taxonomy of Breast Cancer International Consortium (METABRIC) datasets [33] comprising discovery (*n* = 939) and validation (*n* = 845) cohorts, we generated a CCR7–PDPN score based on combined mRNA expression levels. As METABRIC profiles are derived from tumour cores that may include stromal and immune cells, both compartments could contribute to the observed associations. However, neither CCR7 nor PDPN showed strong correlation with general immune (PTPRC) or mesenchymal (ACTA2) markers, and their combined score correlated only weakly (data not shown).

**Fig. 6:**
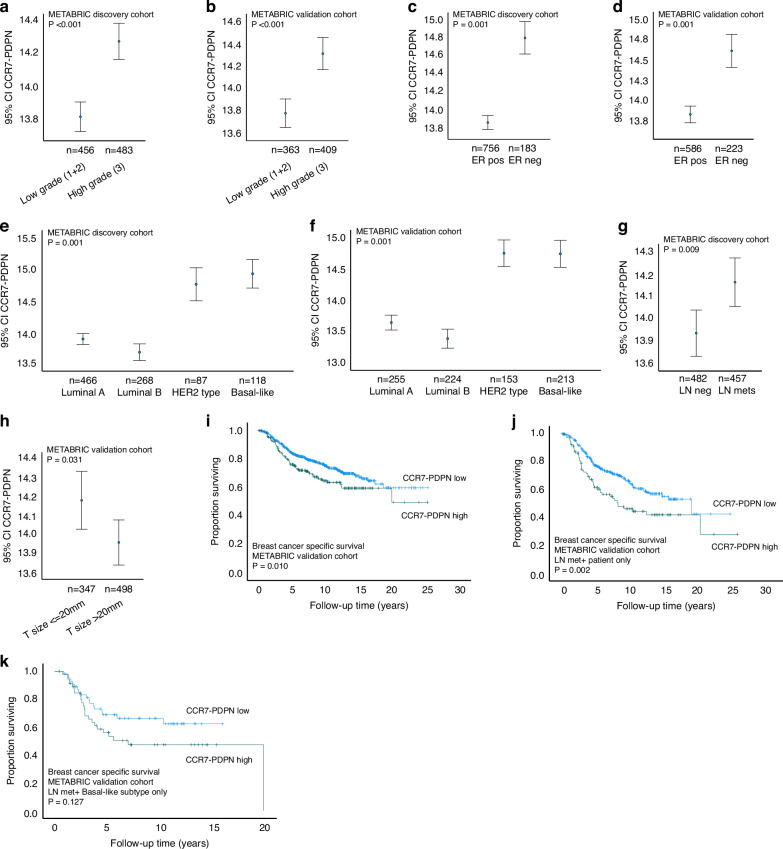
CCR7-PDPN expression across clinicopathological variables and its association with breast cancer-specific survival in the METABRIC cohort. CCR7-PDPN score across histologic grade (**a**, **b**), ER status (**c**, **d**), and molecular subtypes (**e**, **f**) in METABRIC discovery and validation cohorts. CCR7-PDPN score across lymph node status (**g**) and tumour size (**h**) in METABRIC discovery and validation cohort, respectively. **i** A high CCR7-PDPN score is associated with shorter disease-specific survival (METABRIC validation cohort). **j** A high CCR7-PDPN score is also significantly associated with shorter survival in lymph node-positive tumours only. **k** A trend of significance was observed in lymph node-positive, basal-like subtype patients. All Kaplan–Meier survival plots from the METABRIC validation cohort. Data shown with error bars representing 95% confidence interval of the mean, and *p* values by the Mann–Whitney *U* test.

To evaluate how the high CCR7-PDPN score (cut-point upper quartile) was related to tumour phenotypes, common associations in both discovery and validation groups included high histologic grade (*p* < 0.001, Fig. 6a, b), ER negativity (*p* = 0.001, Fig. 6c, d), and aggressive molecular phenotypes (*p* = 0.001, Fig. 6e, f). Additionally, we demonstrated associations with LN metastases (METABRIC discovery cohort, *p* = 0.009, Fig. 6g), and low tumour size (METABRIC validation cohort, *p* = 0.031, Fig. 6h). High CCR7-PDPN expression score is also associated with shorter disease-specific survival (univariate analysis, METABRIC validation cohort, Fig. 6i, Supplementary Table 3A). In contrast, neither CCR7 nor PDPN show independent significant association with shorter disease-specific survival when running the COX univariate and multivariate model with traditional prognostic variables (Supplementary Table 4A, B).

Interestingly, when adding the clinico-pathological variables tumour diameter, histologic grade, ER status and LN status to the multivariate analysis, a high CCR7-PDPN score was demonstrated independently associated with shorter disease-specific survival (METABRIC validation cohort; HR = 1.40, 95% CI: 1.03–1.91, *p* < 0.029, Fig. 6i, Supplementary Table 3A). Moreover, when investigating the prognostic impact of CCR7-PDPN score in LN status met-/met+ separately, our data showed that high CCR7-PDPN score was associated with reduced survival in LN metastasis positive patients (METABRIC validation cohort; HR = 1.81, 95% CI 1.28–2.58, *p* ≤ 0.001, Fig. 6j, Supplementary Table 3B), but not in LN negative patients (data not shown). When investigating the prognostic impact of CCR7-PDPN in individual molecular subtypes, high expression of CCR7-PDPN score demonstrated a trending, significant association with shorter disease-specific survival in LN-positive, basal-like subtype (METABRIC validation cohort; Fig. 6k), but not in the other tumour subtypes.

Together, these data support a correlation between high PDPN and CCR7 and negative prognosis in breast cancer patients.

## Discussion

EO771 TN mammary carcinoma, like most models of cancer in a C57Bl6 background display limited lymphatic metastatic potential [18–20]. We show here that expression of CCR7 enhances in vivo tumour growth and LN metastasis, the latter in line with the long-recognised role of CCR7 in promoting lymphatic dissemination [8]. However, despite high EMT and mesenchymal features of EO771, including the expression of vimentin and low levels of keratins, our data showed that CCR7 expression alone is not sufficient to drive tumour progression and LN metastasis. Instead, a further shift in the mesenchymal phenotype, with PDPN upregulation as a biomarker, is required. The latter was promoted in the hypoxic tumour microenvironment. While PDPN expression was only rarely observed in hypoxic cells, it was consistently detected in clusters of cells adjacent to these regions, reminiscent of what has been described as post-hypoxic tumour cells migrating away from hypoxic areas [46].

That hypoxia can drive changes in the mesenchymal profile, and EMT has been indicated before [47]. It was recently shown to be linked to vascular occlusion, neutrophil extracellular traps with downstream upregulation of TGF-β in hypoxic areas of multiple models of mouse carcinoma and in human cancer [40]. That PDPN can be a biomarker in such a context has previously not been observed, but is supported both by our experimental data and indicated by in silico data from single-cell analysis of human TNBC.

The specific changes, as part of the hypoxia-enhanced mesenchymal-shift and PDPN-expression, or the combination of changes, that are critical for the observed effects on growth and metastasis in CCR7-PDPN EO771, remain to be fully defined, but our data support that immune modulation of the anti-tumour T-cell response is one key change. The growth advantage of CCR7-PDPN EO771 was in line with this notion, abolished in T-cell-deficient hosts. This is also consistent with the fact that TDLNs are major sites for tumour immunity, providing selective pressure for immune-evasive traits to enable metastatic spread to these sites [7].

In EO771, as well as in human TNBC cell lines with high expression of PDPN, baseline interferon responses were suppressed, changes that included downregulation of MHC class I. That cell-intrinsic inhibition of IFN responses can have an inhibitory effect on the anti-tumour response has been shown before [48]. These responses can be triggered by cytosolic nucleic DNA in cancer cells with high genomic instability through damage-associated signalling, such as the cGAS/STING system [49], which has also been associated with tumour dormancy and reactivation [50]. Knockdown of PARP7 (inhibitor of IFN pathways) in EO771 was recently shown to drive increased tumour immunity in vivo, which, like our data support that this tumour model is dependent on immune evasion via IFN pathway suppression [43]. Recently, cell-intrinsic, ligand-independent immune modulatory effects by PDPN were demonstrated for mouse LN fibroblast [51], and direct contribution from PDPN on the tumour cell immune-modulatory phenotype of PDPN-expressing TNBC cannot be excluded. Careful dissection of the relative contributions of PDPN and associated phenotypic changes will require purpose-built models and will be of high interest for future work.

Another possible contributing factor to the promotion of metastasis and tumour growth may be the modulation of tumour-expressed collagens in TNBC with PDPN, effects that were conserved across EO771 and in human TNBC cell lines, as well as in primary tumour enriched for PDPN-expression. Although collagens are mainly associated with cancer-associated fibroblasts, their upregulation in tumour cells has been linked both to proliferation and metastatic niche formation [52, 53]. In EO771 with co-expression of CCR7 and PDPN, the interplay between components of the extracellular matrix and tumour plasticity may contribute to establishing a microenvironment conducive to tumour seeding, growth and metastasis. Further assessment at the protein level and functional evaluation would be required to clarify whether the observed modulation of collagen expression on the mRNA level has a mechanistic role in promoting metastasis or merely accompanies a PDPN-associated tumour state.

Supporting translational relevance, our data show heterogeneous PDPN expression at the protein level in LN metastases. In silico analysis further revealed PDPN in a patient with a metaplastic BC, which is a rare but highly aggressive form of TNBC [54]. Although LN metastasis is generally considered less common in TNBC than in luminal breast cancer, its presence in TNBC is associated with poor prognosis. Moreover, EMT has been linked to nodal metastasis in metaplastic TNBC [55].

While CCR7 has been recognised as a contributor to lymphatic metastasis for over two decades [8], its reliability as a biomarker has remained inconsistent [17]. Our data underscores the combined CCR7–PDPN phenotype as a clinically relevant determinant of aggressive disease. In patient data, a CCR7–PDPN mRNA score was enriched in basal-like tumours and associated with high grade, ER-negativity, and reduced survival, particularly in LN-positive patients. Importantly, the score retained independent prognostic value in multivariate analyses adjusting for tumour size, grade, ER status, and LN status, while either marker alone lacked significance. Although the prognostic impact of the CCR7–PDPN score may reflect contributions from both tumour cells and the stroma, our experimental data demonstrate that PDPN-expressing tumour cells acquire a phenotype characterised by interferon suppression and collagen mRNA upregulation, both of which are linked to immune evasion and progression. Future comprehensive studies resolving the cellular sources of PDPN and CCR7 in patient samples will be of interest. Basal-like and TNBC, including metaplastic TNBC, are candidate subtypes for such studies evaluating the value of PDPN as a biomarker for a metastastatic-permissive tumour state.

The significance of the CCR7–PDPN score in LN-positive patients in the METABRIC dataset could reflect an effect of this phenotype on an increased risk of distant dissemination. In our experimental model, PDPN–CCR7 co-expression promoted lymphatic spread, but we did not observe spontaneous distant macrometastases at the analysed time points. This is consistent with reports that EO771 orthotopic tumours rarely form detectable lung lesions without primary-tumour resection [18, 56]. While our study focused on CCR7-driven lymphatic dissemination, future work should address whether PDPN–CCR7 cooperation also promotes metastasis to distant organs. Beyond LN metastases, CCR7 expression has previously been associated with dissemination to visceral and bone sites [36] and to the brain [57].

Together, our findings identify PDPN as a marker of tumour cell plasticity in TNBC, linked to hypoxia, and reveal the CCR7–PDPN axis as a driver of lymphatic dissemination and a candidate biomarker combination for risk stratification in breast cancer. Further research is warranted to dissect the molecular mechanisms and to validate its prognostic potential in clinical cohorts.

## Supplementary information

Supplementary Tables 1–4

Supp. Figs. 1–11

## Data Availability

Bulk RNA-seq raw data from EO771 is deposited to the NCBI Gene Expression Omnibus database, with access number GSE313218. Code is available from https://github.com/zhi-igp-uu-se/ccr7andpdpn.git. METABRIC gene expression datasets are available at https://ega-archive.org/studies/EGAS00000000083 (Molecular Taxonomy of Breast Cancer International Consortium - METABRIC). Restrictions apply to data generated within this study—these are therefore not publicly available. However, on reasonable request, interested researchers may contact EW and EAH to enquire about access. Request for non-commercial use will be considered and will require a full ethics review.
