# Supplementary material for: Podoplanin-defined tumour plasticity and CCR7-mediated lymphatic metastasis in triple-negative breast cancer
Source: Br J Cancer. 2026 Apr 9;134(12):1730–43. doi: 10.1038/s41416-026-03402-4 (PMC13226698; doi:10.1038/s41416-026-03402-4)
Supplement: Supplementary file 1 — Supplementary Tables 1–4 [file 41416_2026_3402_MOESM1_ESM.pdf]

Supplementary Table 1

The top 100 significantly up- and down-regulated genes across the different E0771 cell derivates - identified using thresholds of log2 fold change >1 (|log2FC| > 1) and adjusted p-value < 0.05 or FDR < 0.05

Downregulation

CCR7 PDPN+ vs X2 PDPN+	CCR7 PDPN- vs X2 PDPN-	X2 PDPN+ vs X2 PDPN-	CCR7 PDPN+ vs X2 PDPN-	CCR7 PDPN+ vs CCR7 PDPN-
Dab2	Glud1	Lgals9	Slpi	Abcg2
Nt5e	Spp1	Oas1a	L1cam	Ly6e
Shld2	Lgals3	Dtna	Lims2	Ifit2
Glud1	Angptl2	Aif1l	Fam171b	Dtna
Tgfbi	Tes	Rigi	Glud1	Cyfip2
Lgals3	Shld2	Ifit2	Shld2	Clec1a
Tfdp1	Slpi	Oas3	Ly6e	Car12
Emb	Stim1	H2-K1	Ifit2	Oas1a
Acta2	Lims2	L1cam	Csrp1	Hivep3
Ly6e	Gpnmb	Serpinb9b	Fam107b	Oas2
Slc39a14	Serpine1	Snx10	Pla1a	Ifi27l2a
Gpnmb	Ltbp1	Bst2	Ccn1	Bicd1
Slpi	Pla1a	Adgrg1	Lipa	Snx10
Spp1	Eno3	Fam107b	C3	L1cam
Nptx1	C3	Irf7	Itgb7	Rabgap1l
Shank2	Camk2n2	Oas2	Krt80	Ablim1
Pla1a	Socs4	Slfn2	Atp6v1b2	Nsd1
Mapk1ip1l	Ereg	Ifi27l2a	Plat	Otud1
Mt2	Plat	Ifi44	Fam149a	Ank2

Ecm1	Epb41l4a	Rtp4	Nectin2	C1s1
Ly75	Fam107b	Apol9a	Oas1a	Acta2
Ereg	Rnf43	Nlrc5	Tfdp1	Gm13147
Creb5	Ubash3b	Tmcc3	Slc39a14	Bche
Pla2g7	St3gal1	Isg15	Esd	Apol9a
Igsf3	Pde4b	Zfand2a	Ccl2	Apol9b
Loxl2	P2rx7	Xaf1	Gpnmb	Oas3
Adamtsl4	Cc2d1a	Car13	Snx10	Tmsb4x
Prdm1	Crybg1	Irf9	Abcg2	Chn1
Ltbp1	Pstpip2	Parp9	Lgals9	1700025G04Rik
Slc44a1	Ly75	Dhx58	Ltbp1	Fam171b
Sardh	Osbp2	Lipa	Stim1	Ifi44
C1s1	Sgms2	Irgm1	Bst2	Adh7
Serpine1	Rgs16	Otud1	Dtna	Shank2
Camk2n2	Plau	Apol9b	Socs4	Lipa
Adcy6	Creb5	Oasl2	Lgals3	Casp4
Socs4	Stard8	Cystm1	Osbp2	Aig1
Gas7	Rnf144b	Ndrg4	H2-K1	Pex2
Stard8	Prrx1	Fam171b	Ly75	Fam107b
Gstm1	Wdhd1	Nrg2	Car12	Rhd
Prrx1	Lipa	Ifih1	Creb5	Thbs2
Ubash3b	Fhdc1	Usp18	P2rx7	Ptpn7
Fam149a	Napsa	Parp14	Cd68	Slfn2
Pde4b	Retreg1	Gdf15	Shank2	Il7
Fhdc1	Cfap45	Ube2l6	Bmpr1a	Adamts6
Hivep3	Itgb7	Cd68	Telo2	Cmklr1

C3	Ndrg4	Apobec1	Serpine1	Ly9
Napsa	Rnasel	Oas1g	Il1rap	Atf3
Deptor	Gls2	Gbp3	St3gal1	Ifih1
Lrrc8c	Pnp	Ccdc88c	Rnf144b	Rtp4
Rassf2	Prmt2	Tent5b	Otud1	Rnd1
1700025G04Rik	Cd68	Phf11d	Slfn2	Akap6
Prtn3	Ap3m2	Panx1	Lrrc8c	Adamts7
Wdhd1	Krt80	Lcp1	Rabgap1l	Map2k6
Abcg2	Plekhh2	Bicd1	Serpinb9b	Pmaip1
Rcn3	Loxl2	Pmaip1	Tap1	Afp
Ccl2	Adamtsl4	Trim30a	Epb41l4a	Ccn2
Rnf43	Tm4sf1	Rnasel	Ubash3b	Col9a1
Itga1	Kyat3	Adamts7	Stard13	Prrx1
Plat	Prdm1	Mtm1	C1s1	Oas1g
Samd9l	Suox	Erc2	Hivep3	Hpse
Igfbp6	Bmp7	Pramel13	Rigi	Irf7
Cacng7	Stab1	Enkur	Prrx1	Apobec1
St3gal1	Shank2	Oas1b	Chn1	Sp100
Slc22a23	Slfn2	Trpv3	Camk2n2	Inpp4b
Oplah	Rtp4	Ddb2	Stard8	Scarf1
Gja4	Gja4	Hsh2d	Oas3	Cdkl5
Col9a1	Irf9	Cd274	Irf9	Hsd17b11
Cyfip2	Eepd1	Clip4	Oas2	Pde5a
Chn1	Vasn	H2-M3	Ereg	Trim46
Osbp2	Scel	Gm8995	Plcxd2	Pde4d
Casp4	Ccl7	Cmpk2	Rtp4	Pramel13

Atp1a2	Armcx1	Tmem71	Cln5	Shroom4
Sgk1	H2-DMa	Trim21	Oasl2	Pax2
Car12	Tbx4	Psmb9	Loxl2	Me3
Plekhh2	Sall2	Serpinb6b	Tm4sf1	Amigo2
Hand1	Adgrg3	Med12l	Ifi44	Atf5
Ly6a	Klf8	Map2k6	Irf7	Gdap1l1
Sall2	Lyz2	Mboat1	Casp4	Msra
Prickle2	Oasl2	Klra4,Klra18	Kyat3	Elmo1
Sez6l2	Gm13387	Igtp	Pde4b	Casp12
Kcnip3	Tgfb2	Tbc1d2	Samd9l	Tbx4
Bmp7	Fabp4	2310034O05Rik	Ifi27l2a	Usp18
Fbxo32	Ero1b	Lin28b	Eno3	Xaf1
Cyp39a1	Hspa12a	Elmo1	Acta2	Ubash3b
Actg2	Lacc1	Nnmt	Sash1	Trim30a
Lyz2	Phf11d	Sdr42e1	Ank2	Nup210
Tm4sf1	Lmo7	Them4	Mcf2l	Lrrc4b
Erbb3	Evi2a	Acot2	Tubb3	Creb5
Itgb3	Isg15	Angptl6	Sgms2	Parp14
Adh7	Ly6a	Cfap58	Car13	Esr1
Hopx	Ttc39c	Rasgrp3	Itga7	Tm4sf1
Lims2	Pcdh10	Klhdc1	Isg15	Plb1
Gli2	Podnl1	Ifi211	Ndrg4	Hfe
Gm13387	Dhh	Oas1c	Wdhd1	Oasl2
Lrrc15	Dhx58	Ppp1r15a	Stab1	Pbx1
Rgs16	Psg22	Trim34a	Rnasel	Brme1
Ifit2	Casp4	Pwwp3b	Ghitm	4632415L05Rik

Slco2b1	Dmxl2	Lmo7	Nt5e	Pced1b
Ttll7	Ckb	Trim12c	Ifih1	Gsap
Pcbp3	Sema3g	Myo18b	Ccn2	Rsad2

Upregulation

CCR7 PDPN+ vs X2 PDPN+	CCR7 PDPN- vs X2 PDPN-	X2 PDPN+ vs X2 PDPN-	CCR7 PDPN+ vs X2 PDPN-	CCR7 PDPN+ vs CCR7 PDPN-
Afg3l1	Ddx19a	Atp1a2	Eya2	Col6a3
Tcf25	Sf3b3	Col3a1	Col3a1	Mylk
Ccr7	Aars	Col6a2	Cmip	Pdpn
Prmt7	Rfwd3	Tgfbi	Bmf	Bin2
Abcb10	Wwp2	Fut8	Cenpn	Mvd
Nup133	Ccr7	Gsta4	Pskh1	Acat2
Rhou	Ap1g1	Lpin1	Gcsh	Gm13652
Ap1g1	Cyb5b	Selenbp1	Gan	Bcl11b
Wdr59	Utp4	Bmf	Nup133	Tm7sf2
Nip7	E2f4	Gstm1	Hsbp1	Pcdh10
Cfdp1	Zc3h18	S100a4	Ranbp10	Gm6871
Terf2	Usp10	Arrdc3	Nutf2	Hspa12a
Mon1b	Nfatc3	Igf2r	Edc4	Tbc1d8
Terf2ip	Cox4i1	Eya2	Col6a3	Gm43800
Kars	Ddx19b	Nos1	Cfdp1	Evc
Cyb5b	Psmd7	Padi3	Tmem170	Sema6d
Vps4a	Cfdp1	Pla2g7	Chst5	Shank3
Nqo1	Nup133	Serpinf1	Kars	Mageb2
Edc4	Chst5	Mical2	Terf2ip	Trib2
Nfatc3	Mlkl	St3gal5	Mon1b	Cfap45
Ddx19b	Cbfb	Heg1	Kif26a	Btnl7-ps
Aars	Dhx38	Nt5e	Abcc3	Plet1
Bcar1	Urb2	Nlrp4f	Ccr7	Faxc

Utp4	Atxn1l	Col9a1	Cbfb	Gm14124
Slc7a6	Mylk	Olfml3	Ddx19a	Prss42
Zc3h18	Gse1	Kif26a	Ddx19b	Smagp
Psmd7	Sntb2	Sardh	Rfwd3	Chst1
Dhx38	Terf2	Abcc3	Mlkl	Actg2
Hsdl1	Hsdl1	Trerf1	Wdr59	Magea9
Hsbp1	Terf2ip	Stra6	E2f4	Gipr
Ddx19a	Slc7a6os	Pdpn	Fhod1	Nipa1
Ist1	Col3a1	Acss2	Ripor1	Aldoc
Cox4i1	Gan	Mylk	Mvd	Evi2a
Wwp2	Acp5	Oplah	Apobr	1700066B19Rik
Usp10	Tcf7l1	Jup	Cdt1	Vasn
Chmp1a	Eya2	Actg2	Cog4	Fam83a
Dync1li2	Mtss2	Btnl7-ps	St3gal2	Matn4
Atxn1l	6430548M08Rik	Col6a3	Lss	Ryr1
Mlkl	Slc7a5	Lrrc15	Col6a2	Ky
Vac14	Olfm1	A4galt	Cmtm3	Gm49701
Sf3b3	Cog8	Gas7	Cox4i1	Gm16897
Cog4	Tango6	Platr14	Map1lc3b	Gpat2
E2f4	Abcc3	Emilin1	Zcchc14	Trpm6
Atp6v0d1	Nos1	Cdon	Zc3h18	Gm17455
Ripor1	Dhodh	Rcn3	Uaca	Pfn2
Urb2	Cyba	Sema6d	Nfatc3	Rpl7a-ps5
Nob1	Emc8	Dock4	Slc7a6os	Smad6
Zcchc14	Pskh1	Atp2b4	Hsdl1	Celf2
Rfwd3	Meak7	Vwa5b1	Sntb2	Prrx2

Taf5l	Ankrd11	Gm2381	Vps4a	Stard8
Cenpn	Rnf166	Pcbp3	Cog8	Gm10419
Map1lc3b	Camk1d	Car9	Terf2	Epm2a
Cdt1	Trim2	Aox3	Cyb5b	Lypd5
Cog8	Ube2cbp	Hspa12a	Ist1	Myo15b
Cbfb	Mvd	Obscn	Atxn1l	Gm14547
Cmip	Ctu2	Ttyh3	Ap1g1	Lbx2
Slc7a6os	Adat1	Cacng7	Mylk	Tnni3
St3gal2	Scarf1	Fxyd1	Vac14	Syt8
Ranbp10	Pop1	Itpr2	Sf3b3	Ankrd23
Nae1	Tmsb4x	Hmgcs1	Cyp51	Gm14231
Fbxo31	Pdpr	Cyp39a1	Meak7	Dact1
Gcsh	Tmem170	Slit1	Usp10	Tex101
Gse1	Afp	Rassf2	6430548M08Rik	Akr1c13
Spg7	Col6a3	Stard10	Gse1	Pcyt1b
Nutf2	Fhod1	Hand1	Wwp2	Gm12648
Mylk	Taf1c	Dpep1	Psmd7	Uroc1
6430548M08Rik	Phlpp2	Lrrc32	Dhx38	Cyp46a1
Atmin	Ccsap	Gpsm3	Nqo1	Rpl10-ps3
Mtss2	Gsta4	Gm47223	Trim2	Angpt2
Thap11	Pcdhgb1	Atp2c2	St3gal4	Gm5617
Mphosph6	Glg1	Acat2	Tcf25	Gck
Aprt	Anxa6	Txnip	Atmin	Gm10175
Tango6	Trappc2l	Man1c1	Rnf166	Gm5619
Sntb2	Platr14	Trib2	Acp5	Rps13-ps4
Tmem170	Bmf	Siglec1	Phlpp2	Orm3

Acd	Klhdc4	Bin2	Dhodh	Gm12203
Cmtm3	Mttp	Acacb	Atp1a2	Large2
Rnf166	Osgin1	Map1a	Nos1	Frmd4b
Dhodh	Nlrp4f	Slco2b1	Tango6	Rps12-ps4
Piezo1	Srl	Plec	Lpin1	5830448L01Rik
Chst5	Acot11	Ube2cbp	Olfm1	
Trim2	Gins2	Prex1	Gas8	
Phaf1	Cyp2j6	Fam83h	Galns	
Meak7	Tmem208	Aldh3a1	Gsta4	
Zfpm1	4930461G14Rik	Disp2	Etv1	
Ctu2	Gm13147	Gm48284	Mtss2	
Rab4a	Serpinf1	Pcdh10	Klhdc4	
Mvd	2810013P06Rik	Lbp	Fcsk	
Cmtm4	Mthfsd	Gm7967	Ctu2	
Gas8	Txnl4b	Nfatc2	Acot11	
Galns	Bche	Egr3	Padi3	
Mthfsd	Rbpms2	Fndc5	Gins2	
Slc12a4	Psd2	Tbc1d8	Acd	
Slc39a10	Pigp	Gmpr	Mphosph6	
Col6a3	Foxl1	Mdga1	Atp2c2	
Taf1c	Kif26a	Pla2g5	Nsdhl	
Klhdc4	Padi3	Magea10	Nlrp4f	
Gan	Mlycd	Inava	Enkd1	
Spire2	Vwa5b1	Zfp978	Mthfsd	
Fhod1	Hsf4	Egr1	Cyria	

Supplementary Table 2. Overview of Antibodies

Flow cytometry

Antibody	Clone	Company	Dilution
PerCP-Cy5.5 anti-CD11b	M1/70	Thermo Fisher Scientific	1:50
PerCP-Cy5.5 anti-CD45	30-F11	Thermo Fisher Scientific	1:50
BV421 anti-Ter119	TER-119	BD Biosciences	1:50
eFluor 450 anti-Ter119	TER-119	Thermo Fisher Scientific	1:50
eFluor 660 anti-Podoplanin	eBioB.1.1	Thermo Fisher Scientific	1:50
PE-Cy7 anti-CD31	390	Thermo Fisher Scientific	1:50
APC anti-CCR7	4B12	Thermo Fisher Scientific	1:50

Immunofluorescence — Primary antibodies for mouse antigens (1°)

Antibody	Clone	Company	Dilution
Anti-mouse IgD-FITC	11-26c	Thermo Fisher Scientific	1:50
Anti-mouse IgM-FITC	11/41	Thermo Fisher Scientific	1:50
anti-tdTomato (Goat polyclonal)	polyclonal	Sicgen	1:200
AF647 / FITC anti-CD21/CD35	7E9	BioLegend	1:50
Anti-LYVE1 (Rabbit polyclonal)	polyclonal	ReliaTech	1:200
Anti-CD169	MOMA-1	BioRad	1:100

CD3e-FITC	eBio500A2	Thermo Fisher Scientific	1:50
Anti-PDGFRβ	28E1	Cell Signaling	1:50
anti-Podoplanin-eFluor 660	eBioB.1.1	eBioscience / Thermo Fisher Scientific	1:50
Anti-CD8	53-6.7	BioLegend	1:50
Anti-FITC-488 (Mouse monoclonal)	RRID: AB_2339038	Jackson ImmunoResearch	1:300

Antibodies for human antigens — Primary antibodies (1°)

Antibody	Clone	Company	Dilution
PDPN	D2-40	Dako / Agilent	1:50
Cytokeratin 18 (goat polyclonal)	polyclonal	Abcam	1:20
PDGFRβ	28E1	Cell Signaling	1:50

Secondary antibodies (2°)

Antibody	Clone	Company	Dilution
anti-Rabbit AF647	Donkey polyclonal	Thermo Fisher Scientific	1:300
anti-Goat AF555	Donkey polyclonal	Thermo Fisher Scientific	1:300
anti-Rabbit AF488	Donkey polyclonal	Thermo Fisher Scientific	1:300
anti-Rabbit AF488	Donkey polyclonal	Jackson ImmunoResearch	1:300
anti-Goat AF555	Donkey polyclonal	Thermo Fisher Scientific	1:300
anti-Rat AF488	Donkey polyclonal	Jackson ImmunoResearch	1:300
anti-Mouse AF594	Donkey polyclonal	Thermo Fisher Scientific	1:300

Supplementary Table 3: Univariate and multivariate survival analyses.

3A

METABRIC validation cohort, All subtypes					
**Variables**	**n**	**Univariate HR (95% CI)**	**p**	**Multivariate (95% CI)**	**p**
**Tumor diameter**					
<2.0 cm	347	1		1	
≥2.0 cm	498	2.00 (1.49-2.67)	**<0.001**	1.94 (1.42-2.63)	**<0.001**
**Histologic grade***					
1+2	363	1		1	
3	409	1.66 (1.25-2.20)	**<0.001**	1.23 (0.89-1.68)	**NS**
**Nodal status**					
Negative	440	1		1	
Positive	405	2.86 (2.14-3.80)	**<0.001**	2.34 (1.73-3.15)	**<0.001**
**ER status****					
ER positivity	586	1		1	
ER negativity	223	1.76 (1.33-2.32)	**<0.001**	1.48 (1.10-2.00)	**0.008**
**CCR7-PDPN score*****					
Low expression	634	1		1	
High expression	211	1.45 (1.09-1.93)	**0.010**	1.40 (1.03-1.91)	**0.029**

*Missing: 73 **Missing: 36 ***Cut-point Q1-3 low CCR7-PDPN score Q4 high CCR7-PDPN score
n= number of patients; HR: Hazard Ratio; CI: Confidence interval; P: p-values. NS: Not significant. Cox regression analysis (backward stepwise model

3B

METABRIC validation cohort, All subtypes, LN met+ only					
**Variables**	**n**	**Univariate HR (95% CI)**	**p**	**Multivariate (95% CI)**	**p**
**Tumor diameter**					
<2.0 cm	138	1		1	
≥2.0 cm	267	1.82 (1.26-2.62)	**0.001**	1.97 (1.34-2.89)	**<0.001**
**Histologic grade***					
1+2	162	1		1	
3	220	1.48 (1.04-2.09)	**0.028**	1.16 (0.79-1.70)	NS
**ER status****					
ER positivity	279	1		1	
ER negativity	124	1.45 (1.03-2.03)	**0.029**	1.19 (0.81-1.74)	NS
**CCR7-PDPN score*****					
Low expression	302	1		1	
High expression	103	1.70 (1.21-2.38)	**0.002**	1.81 (1.28-2.58)	**<0.001**

*Missing: 23 **Missing: 2 ***Cut-point Q1-3 low CCR7-PDPN score Q4 high CCR7-PDPN score
n= number of patients; HR: Hazard Ratio; CI: Confidence interval; P: p-values. NS: Not significant. LN Met+: Lymph node metastasis positive. Cox regression analysis (backward stepwise model)

(Cox' proportional hazards regression) with death from breast cancer as end-point. All subtypes (**A**: METABRIC validation cohort, n=845) and all subtypes, lymph node metastasis only (**B**: METABRIC validation cohort, n=405).

Følsomhet Intern (gul)

Supplementary Table 4: Discovery and Validation cohort, ALL subtypes: Uni- and multivariate analysis

4A

**METABRIC Discovery cohort, All subtypes**					
**Variables**	**n**	**Univariate HR (95% CI)**	**p**	**Multivariate (95% CI)**	**p**
**Tumor diameter**					
<2.0 cm	409	1		1	
>2.0 cm	530	2.02 (1.54-2.64)	**<0.001**	1.51 (1.14-2.01)	**0.004**
**Histologic grade**					
1+2	456	1		1	
3	483	1.86 (1.44-2.42)	**<0.001**	1.54 (1.18-2.01)	**0.001**
**Nodal status**					
Negative	482	1		1	
Positive	457	2.49 (1.91-3.24)	**<0.001**	2.08 (1.58-2.73)	**<0.001**
**CCR7**					
	939	1.08 (0.94-1.25)	0.246	0.97 (0.83-1.13)	0.748

n= number of patients; HR: Hazard Ratio; CI: Confidence interval; P: p-values.

**METABRIC Discovery cohort, All subtypes**					
**Variables**	**n**	**Univariate HR (95% CI)**	**p**	**Multivariate (95% CI)**	**p**
**Tumor diameter**					
<2.0 cm	409	1		1	
>2.0 cm	530	2.02 (1.54-2.64)	**<0.001**	1.51 (1.14-2.01)	**0.004**
**Histologic grade**					
1+2	456	1		1	
3	483	1.86 (1.44-2.42)	**<0.001**	1.54 (1.18-2.01)	**0.001**
**Nodal status**					
Negative	482	1		1	
Positive	457	2.49 (1.91-3.24)	**<0.001**	2.08 (1.58-2.73)	**<0.001**
**PDPN**					
	939	1.08 (0.91-1.29)	0.347	1.09 (0.92-1.30)	0.297

n= number of patients; HR: Hazard Ratio; CI: Confidence interval; P: p-values.

4B

METABRIC Validation cohort, All subtypes					
**Variables**	**n**	**Univariate HR (95% CI)**	**p**	**Multivariate (95% CI)**	**p**
**Tumor diameter**					
<2.0 cm	347	1		1	
>2.0 cm	498	2.00 (1.49-2.67)	**<0.001**	1.85 (1.36-2.51)	**<0.001**
**Histologic grade***					
1+2	363	1		1	
3	409	1.66 (1.25-2.20)	**<0.001**	1.41 (1.05-1.88)	**0.021**
**Nodal status**					
Negative	440	1		1	
Positive	405	2.86 (2.14-3.80)	**<0.001**	2.32 (1.73-3.12)	**<0.001**
**CCR7**					
	845	1.07 (0.94-1.21)	0.262	1.03 (0.91-1.18)	0.567

*missing 73. n= number of patients; HR: Hazard Ratio; CI: Confidence interval; P: p-values.

METABRIC Validation cohort, All subtypes					
**Variables**	**n**	**Univariate HR (95% CI)**	**p**	**Multivariate (95% CI)**	**p**
**Tumor diameter**					
<2.0 cm	347	1		1	
>2.0 cm	498	2.00 (1.49-2.67)	**<0.001**	1.84 (1.35-2.49)	**<0.001**
**Histologic grade***					
1+2	363	1		1	
3	409	1.66 (1.25-2.20)	**<0.001**	1.43 (1.07-1.90)	**0.014**
**Nodal status**					
Negative	440	1		1	
Positive	405	2.86 (2.14-3.80)	**<0.001**	2.33 (1.73-3.13)	**<0.001**
**PDPN**					
	845	1.03 (0.87-1.21)	0.722	1.04 (0.87-1.24)	0.624

*missing 73. n= number of patients; HR: Hazard Ratio; CI: Confidence interval; P: p-values.

(Cox' proportional hazards regression) with death from breast cancer as end-point. All subtypes. **A**: METABRIC discovery cohort, n=939. **B**: METABRIC validation cohort, n=845.
