# Supplementary material for: Podoplanin-defined tumour plasticity and CCR7-mediated lymphatic metastasis in triple-negative breast cancer
Source: Br J Cancer. 2026 Apr 9;134(12):1730–43. doi: 10.1038/s41416-026-03402-4 (PMC13226698; doi:10.1038/s41416-026-03402-4)
Supplement: Supplementary file 2 — Supp. Figs. 1–11 [file 41416_2026_3402_MOESM2_ESM.pdf]

A

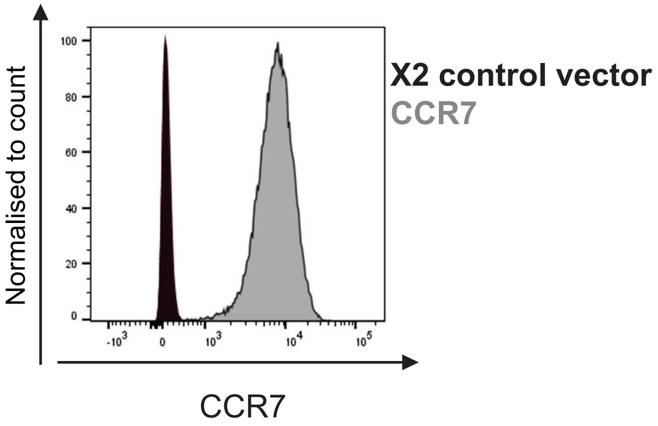

B

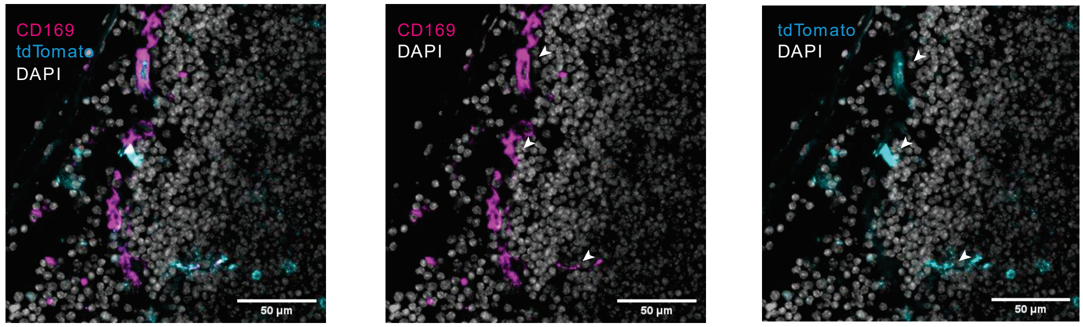

Supplementary Fig. 1: FACS analysis of CCR7 in EO771 and detection of tumour-derived tdTomato in LN macrophages. A) Detection of surface expressed CCR7 by flow cytometry in control (EO771 X2 control vector, black and EO771 CCR7, dark gray). Y-axis normalized to mode. B) Staining of tdTomato (cyan) and CD169 (magenta) subcapsular macrophages, arrows indicate double staining. Scale bar 50 µm (representative of at least 6 TDLNs).

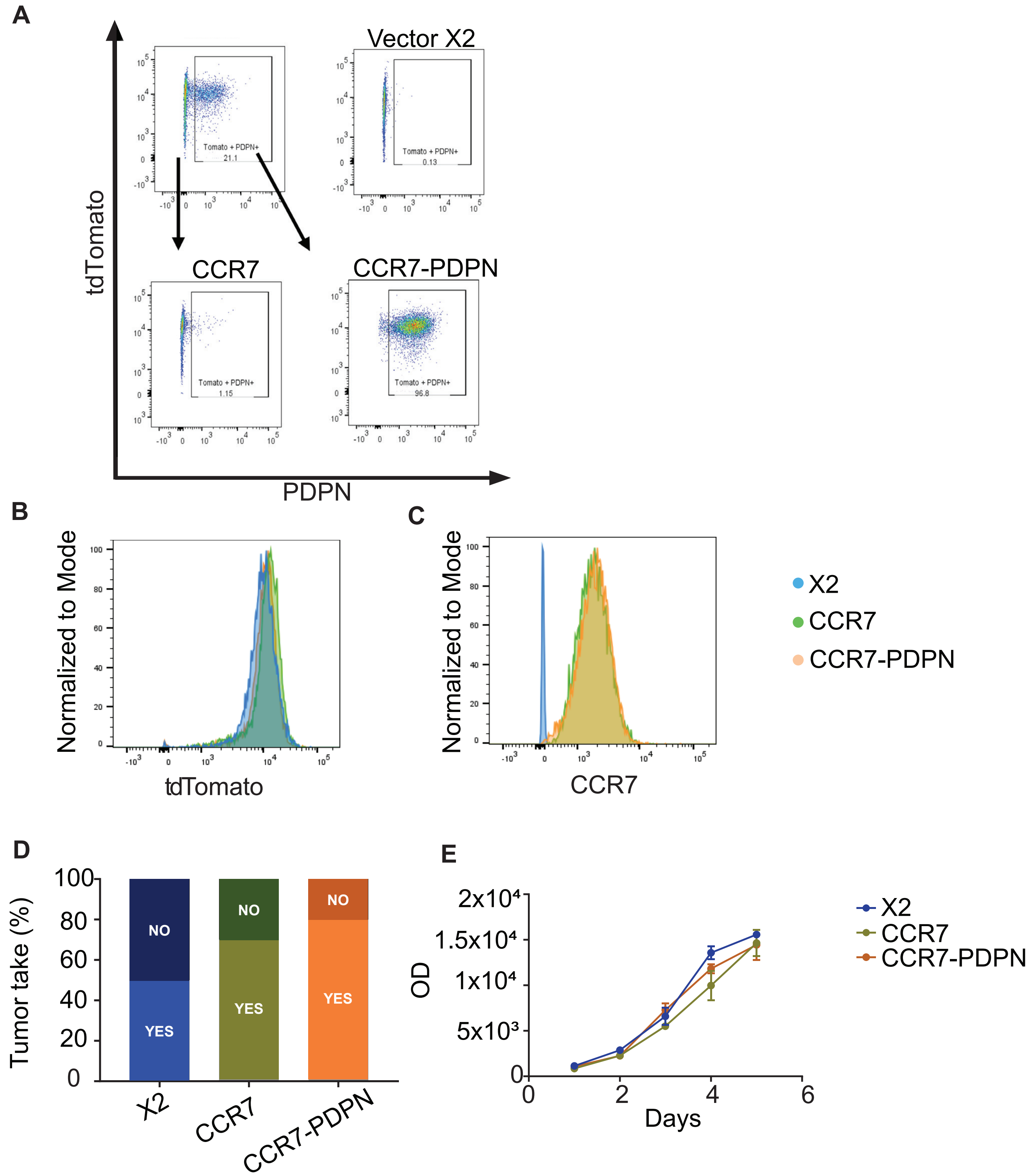

Supplementary Fig. 2: In vitro characterization EO771 with and without CCR7 and PDPN and tumour take in vivo. A) Cell sorting of PDPN positive and negative populations allowed the establishment of stable cell population of EO771 CCR7 with and without PDPN. Plots show representative FACS analysis for expression of tdTomato and PDPN. PDPN is 21,1% in EO771-CCR7, 0.13 % EO771 X2 vector control, 1.15% in EO771-CCR7 selected for PDPN negative cells and 96,5% in EO771 CCR7-PDPN enriched for double positive cells. B) Histogram displaying the average tdTomato expression across the three different cell derivates. C) CCR7 expression is detected in both CCR7 PDPN positive and negative (green and orange) but not X2 PDPN negative cells. D) Tumour uptake across 30 injections in each group (15 mice) 50%, 70% and 80% X2, CCR7, and CCR7-PDPN, respectively. E) In vitro proliferation assay, based on CyQUANT® Cell Proliferation Assay Kit (Invitrogen). Data points indicate the mean values of three replicates per sample. The error bars show SD. Two-way ANOVA shows no significant differences between groups at any time point. .

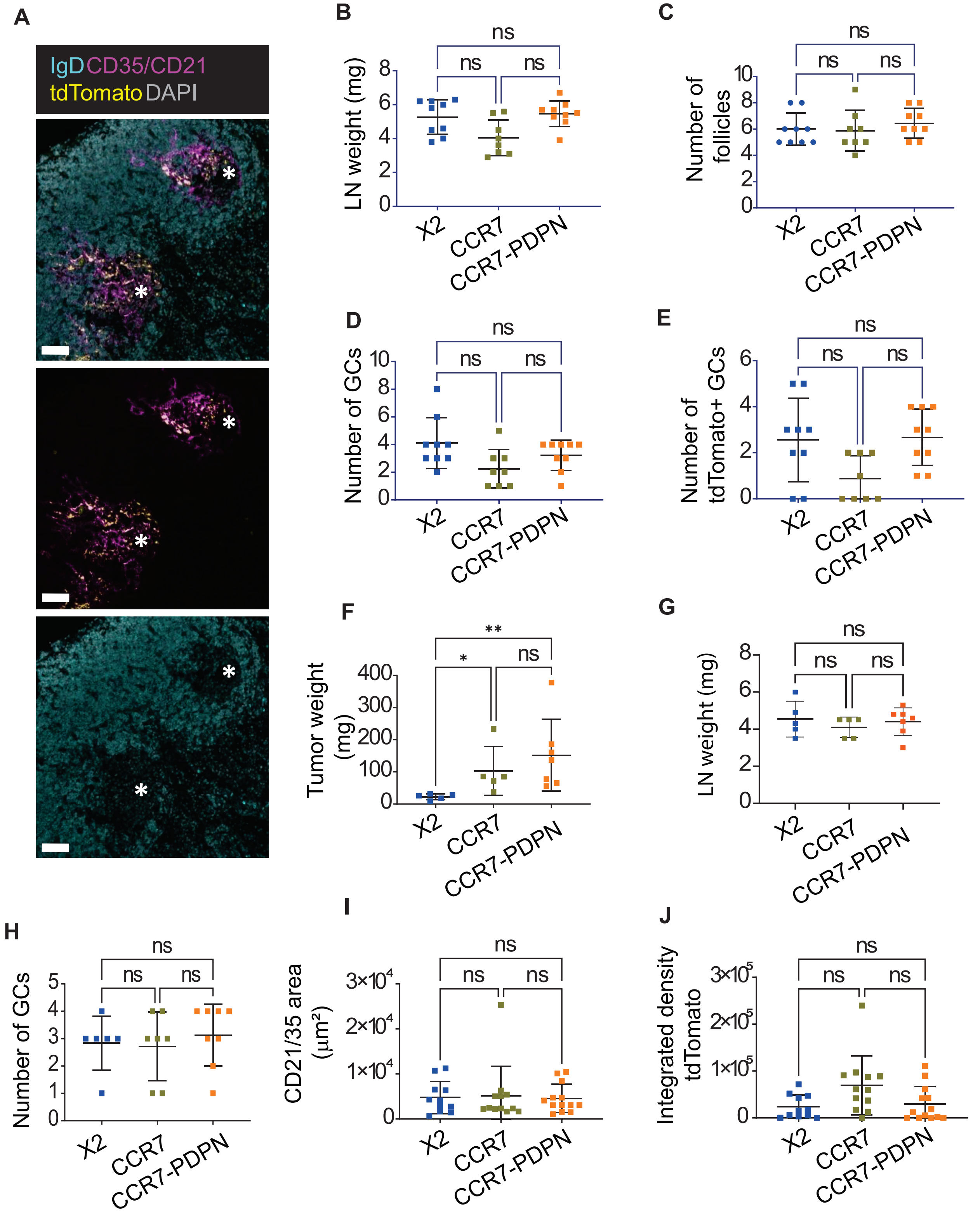

Supplementary Fig. 3: GC formation and tdTomato-positive immunocomplex deposition are similar in EO771 with or without CCR7 and PDPN. A) TDLNs from EO771 CCR7 stained for FDCs (CD21/35), follicular B-cells (IgD), tdTomato and nuclei (DAPI). Scale bar 50 µm. Stars indicate initiated GC formation based on downregulation of IgD in the centre of the B-cell follicle. B-E) Analysis of TDLNs of end-stage tumours. Analysis of the TDLNs was performed in a central section, and consistency across samples is shown by equal number of follicles. B) TDLN size in mg. C) Numbers of follicles in a central section. D) Number of germinal centers (GC). E) tdTomato positive GCs. F-J) Analysis of TDLNs day 14 after tumour injection. F) Weight of tumours in the groups used for analysis. G) Weight of TDLNs on day 14 after tumour injection. H) Number of GCs. I) Analysis FDC (CD21/35) positive area in GC. X2 (n=11), CCR7 (n=12) and CCR7-PDPN (n=13). J). Integrated density for the tdTomato signal was calculated by tdTomato mean grey value x tdTomato area for each GC. No significant differences across groups. Kruskal Wallis Test and Dunn multiple comparison were used.

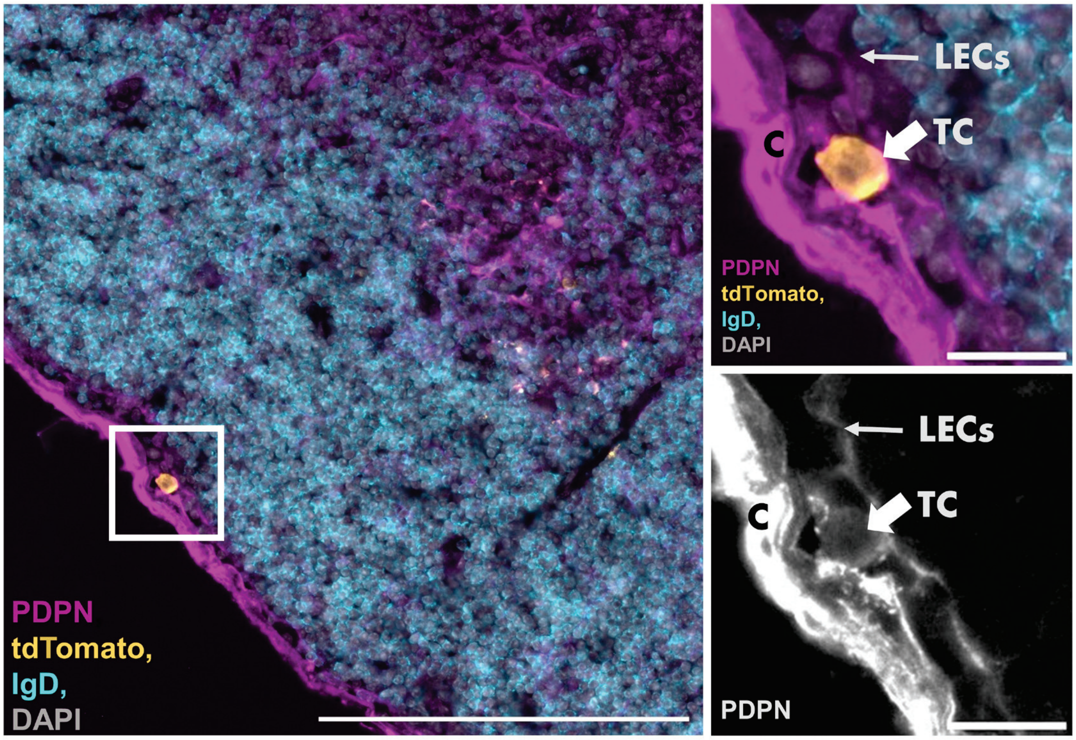

Supplementary Fig. 4: LN metastases from tumours formed from injection of CCR7 positive PDPN negative cells, display PDPN expression. Left overview of LN subcapsular sinus (capsule and LECs outlined by PDPN staining in magenta) with underlying cortex (B-cell zone outlined with staining for IgD in cyan) with a micrometastatic cell within the subcapsular sinus (stained for tdTomato in yellow) and DAPI is shown in grey (nuclei). Insets upper right show capsule (denoted C in figure), floor LECs thin arrow and tumour cell (denoted TC) thick arrow. Inset lower right show single channel PDPN in grey. Scale bar 20 μm.

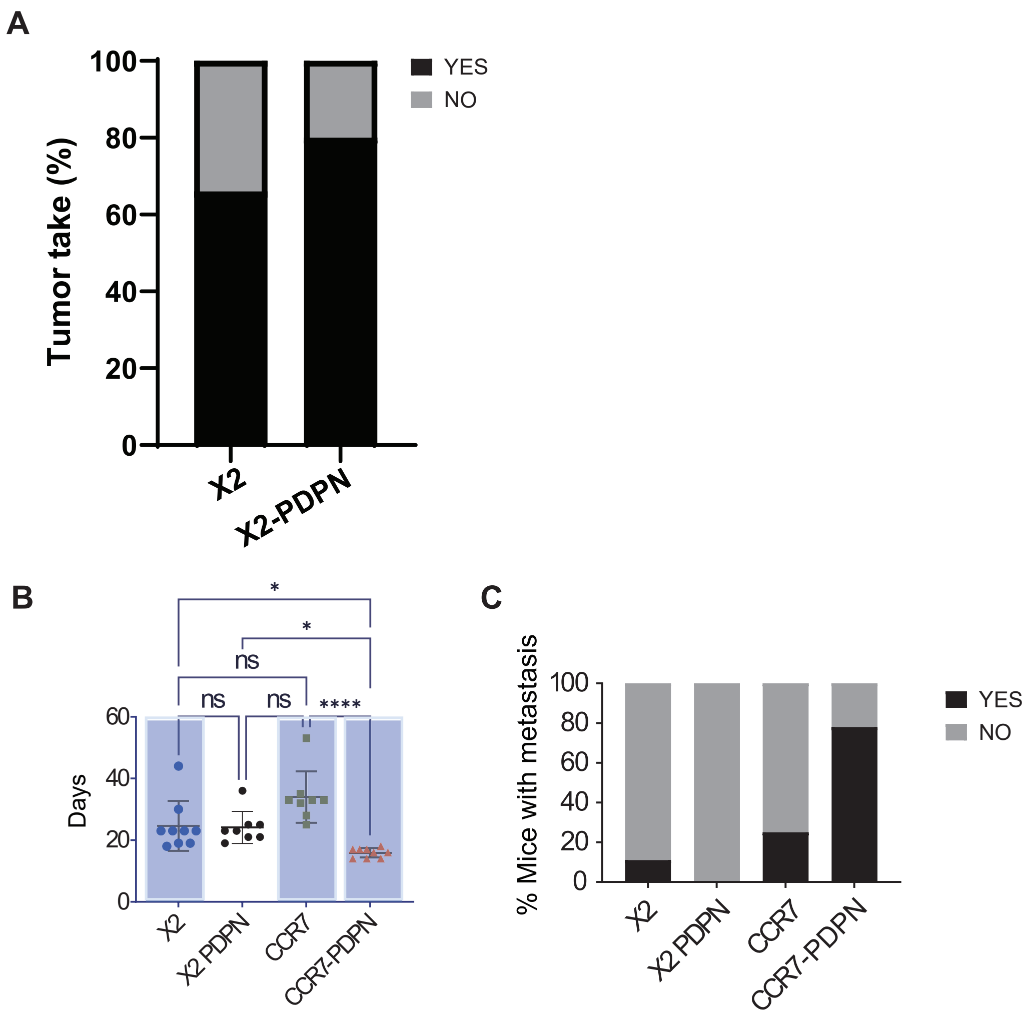

Supplementary Fig. 5: EO771 X2-PDPN characterization. A) Tumour take 65.7% vs. 83% at day 14. n=38 respectively n=30 mice injected across 2 experiments. B-C) X2, CCR7 and CCR7-PDPN same data as shown in Fig. 2H, complemented with X2-PDPN tumours. B) Time after tumour inoculation for samples used for determination of LN metastasis in C). C) LN metastasis was determined by manual assessment of at least 3 levels of each LN (C-D) X2 (n = 9), X2-PDPN (n=8), CCR7 (n = 8), CCR7-PDPN (n = 9).

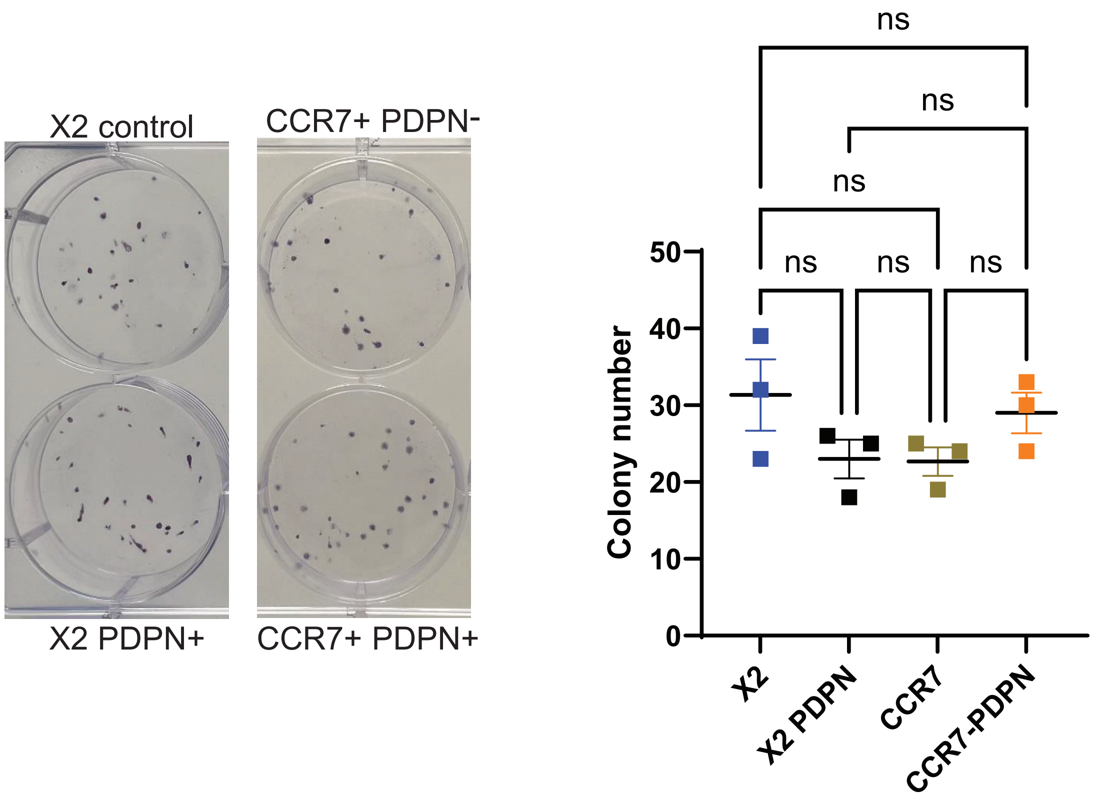

Supplementary Fig. 6: Colony forming assay EO771. Representative images of colony on the left. 100 single cells were seeded in each well of a 6-well plate and were allowed to grow for 14 days. Quantification of colony numbers, on the right, is presented in the scatter plot with standard error of the mean (SEM). No significant differences were observed among the groups (n=3) using the Kruskal-Wallis test with Dunn's multiple comparison test.

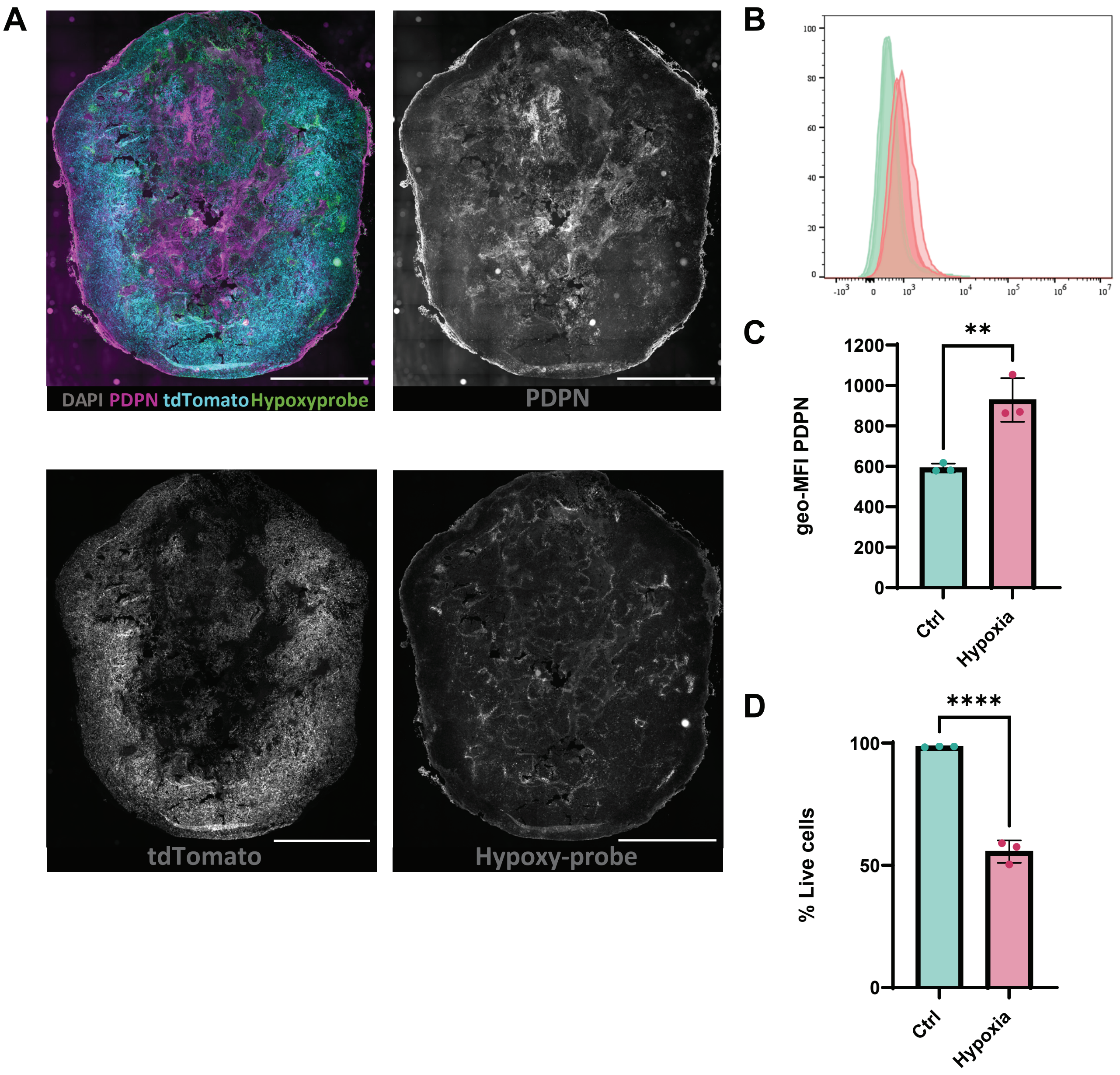

Supplementary Fig. 7: Hypoxy-probe staining. Staining of an EO771-CCR7 tumour for hypoxy-probe (green) together with detection of tdTomato (cyan), PDPN (magenta) Nuclei (DAPI grey). Single channel images shown in grey. Scale bar 2 mm. PDPN is seen in the dense capsule-like rim around the tumour and is induced in the central part of the tumour. Hypoxy-probe stain areas between live and necrotic cells. PDPN staining of necrotic cells is also visible. B) Histogram showing PDPN detection by FACS in EO771 control cells (blue) and EO771 cells cultured for 8 hours with an enzymatic glucose oxidase/catalase system (GOX/CAT; red) for hypoxia. C) Geometric mean fluorescence intensity of PDPN in control versus GOX/CAT-treated cells. D) Percentage of live cells in control and GOX/CAT-treated conditions. C–D) Statistical significance was assessed using an unpaired t-test (**$p < 0.01$; ****$p < 0.0001$).

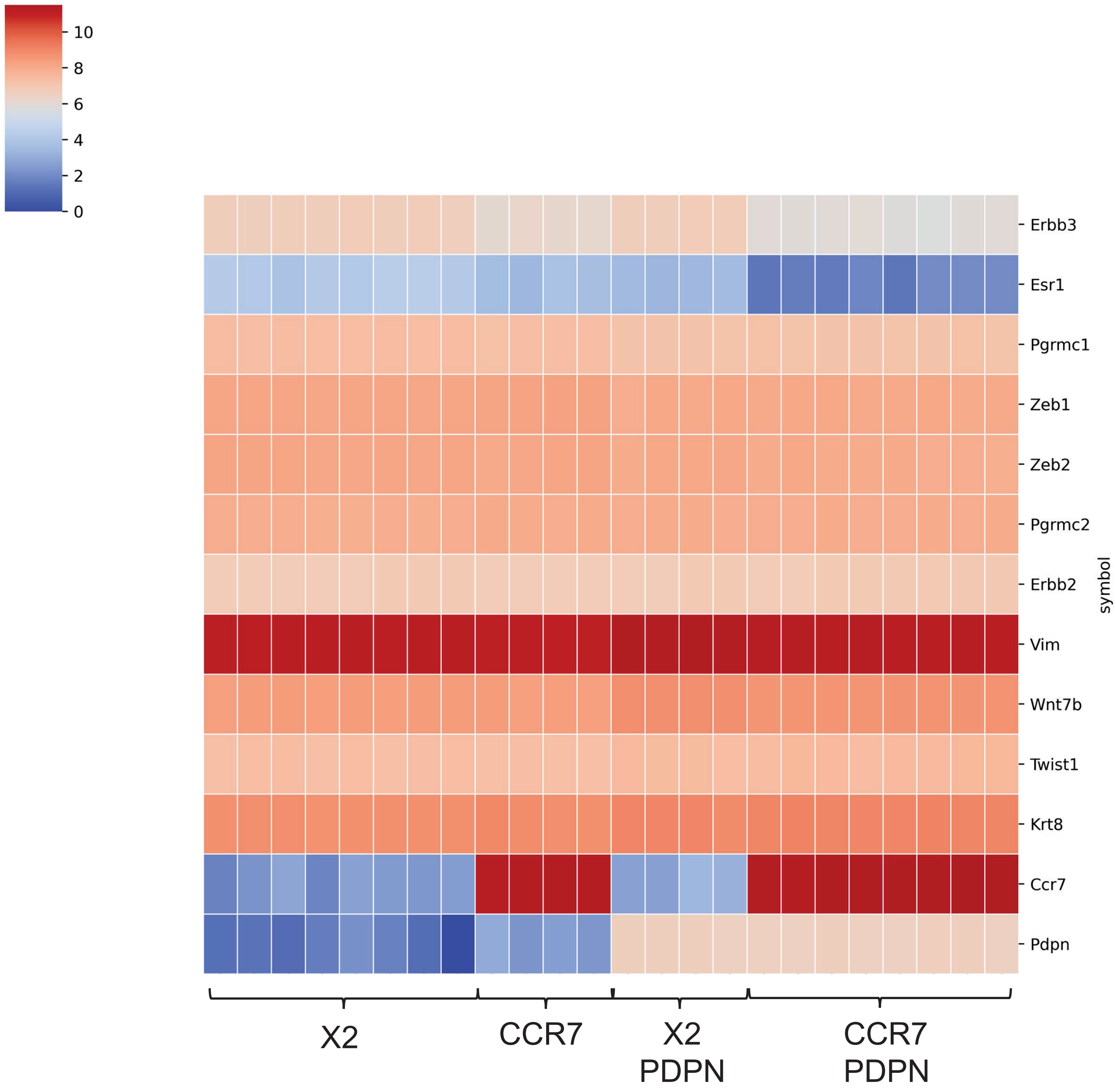

Supplementary Fig. 8: Expression of key genes associated with human breast cancer subtypes and EMT in EO771 Cells with and without CCR7 and PDPN. Heatmap show the expression of selected key genes associated with human breast cancer subtypes and epithelial-to-mesenchymal transition (EMT). Data are presented as normalized counts. Rows represent genes, and columns represent cell derivates (CCR7-positive, PDPN-positive, and corresponding negative controls).

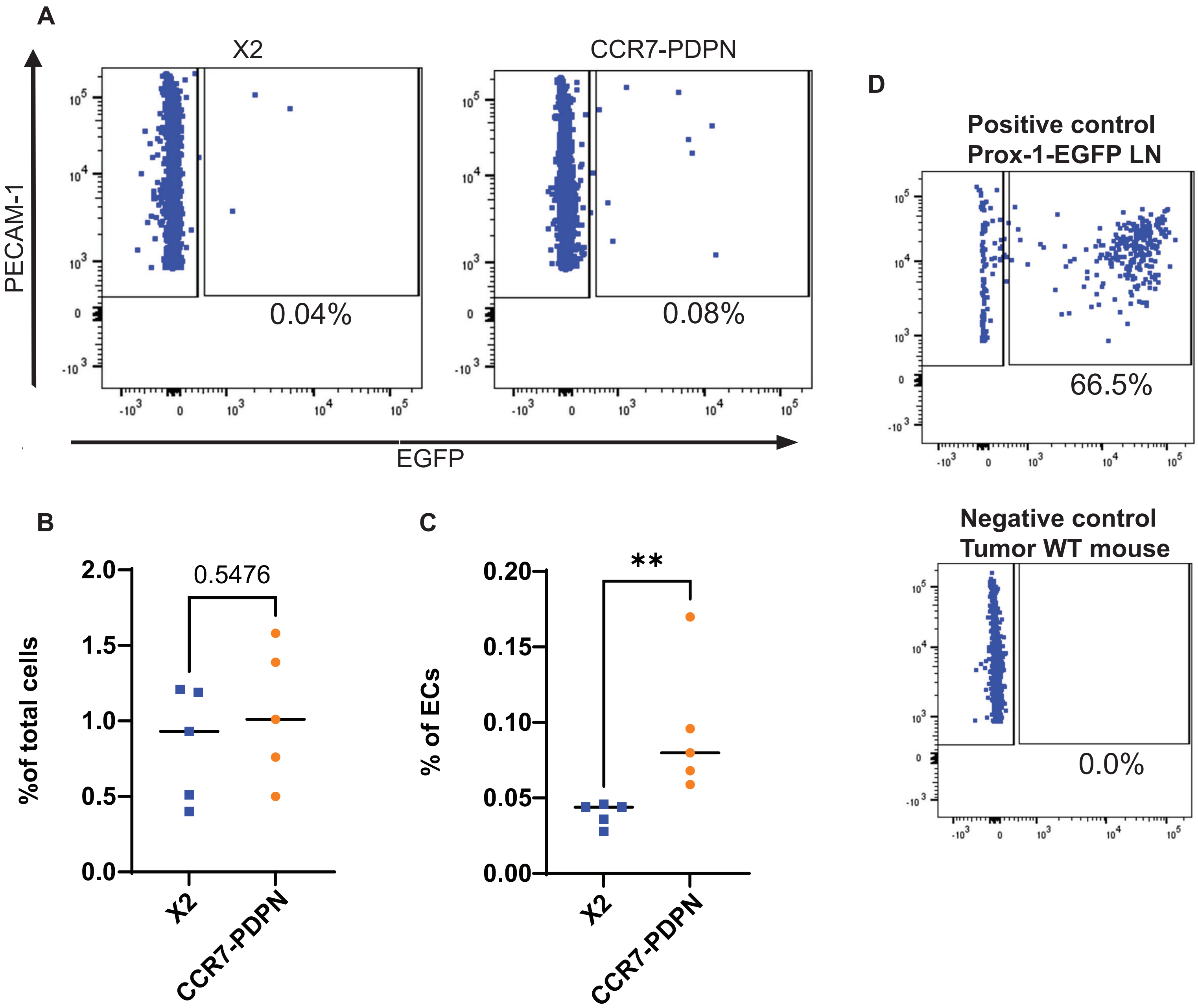

Supplementary Fig. 9: Endothelial cell analysis in tumours. A) Dot-plots of FACS detection of Prox-1-GFP cells within CD45/CD11b negative, PECAM-1 positive endothelial cells, in vector control X2 (left - 0.4% of total EC population) and CCR7-PDPN EO771 tumours (right - 0.8% of total EC population). B) % Prox-1-GFP LECs in X2 vector control and CCR7-PDPN EO771 tumors. ** p< 0.01 n=5 C) % total endothelial cells in in X2 vector control n=5 (B-C Mann Whitney test). D) Positive and negative control for Prox1-EGFP detection: upper LN from Prox-1-EGFP mouse lower detection of EGFP in EC-gate of tumour formed in wild type mouse.

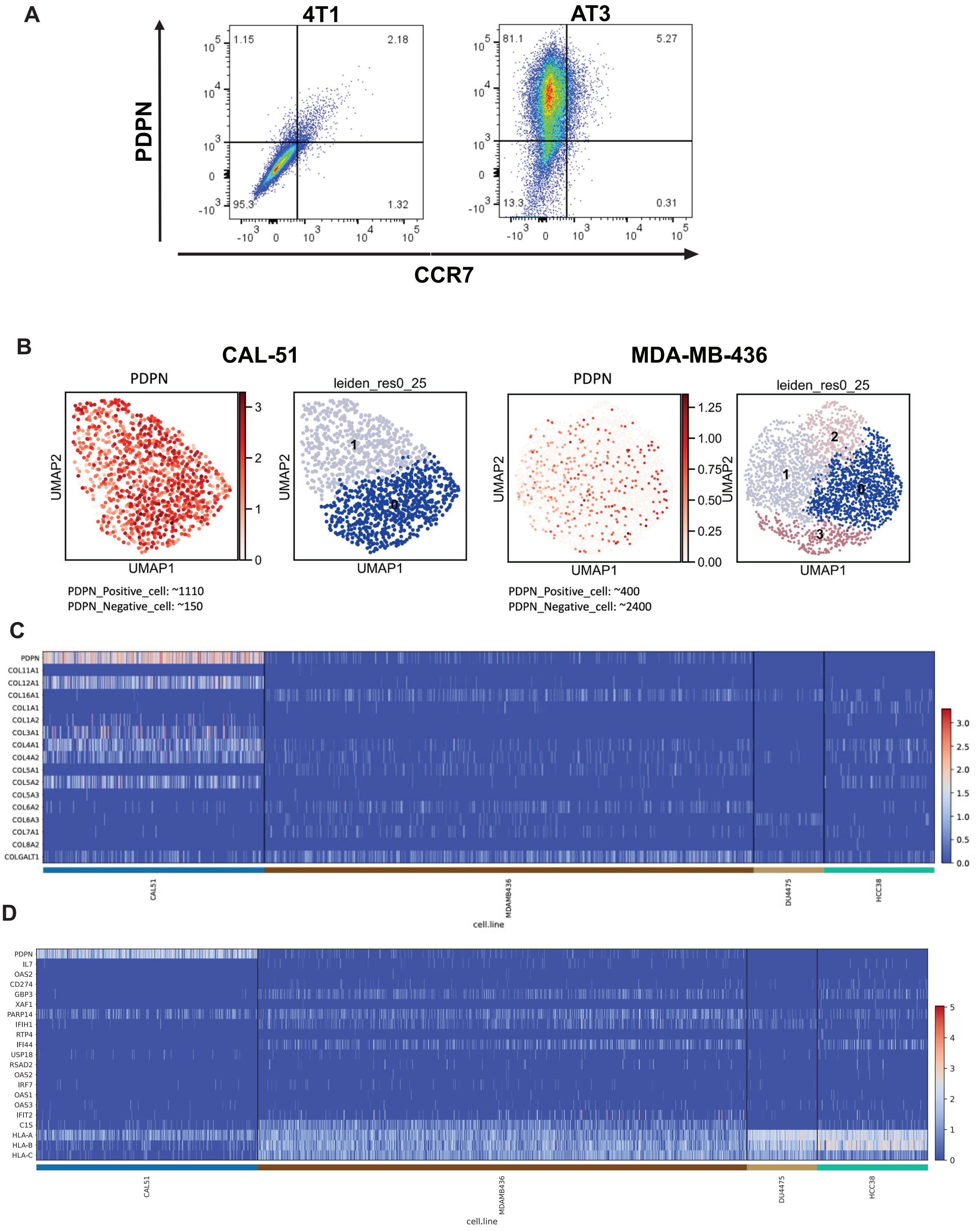

Supplementary Fig. 10: Analysis of mouse mammary and human breast cancer cell lines. A) Dotplots of PDPN and CCR7 expression in 4T1 and AT3 mammary carcinomas analysed by flow cytometry, displaying heterogenous expression of both markers. Representative of 3 repeated experiments. B) Leiden clustering (resolution =0.25) of scRNA-seq data of human TNBC cell lines, left (CAL-51) right (MDA-MB-436) and heatmap across the UMAP space for PDPN expression, with corresponding cell counts added for information. C-D) Z-scores of normalized gene expression, where Z-score = 0 represents the mean gene expression. Gene names are indicated on the left side of the heat maps for reference. C) EMT-related collagens in PDPN positive and PDPN-negative TNBC cell lines. D) IFN-related and immune-related genes (i.e., *IL-7*).

A

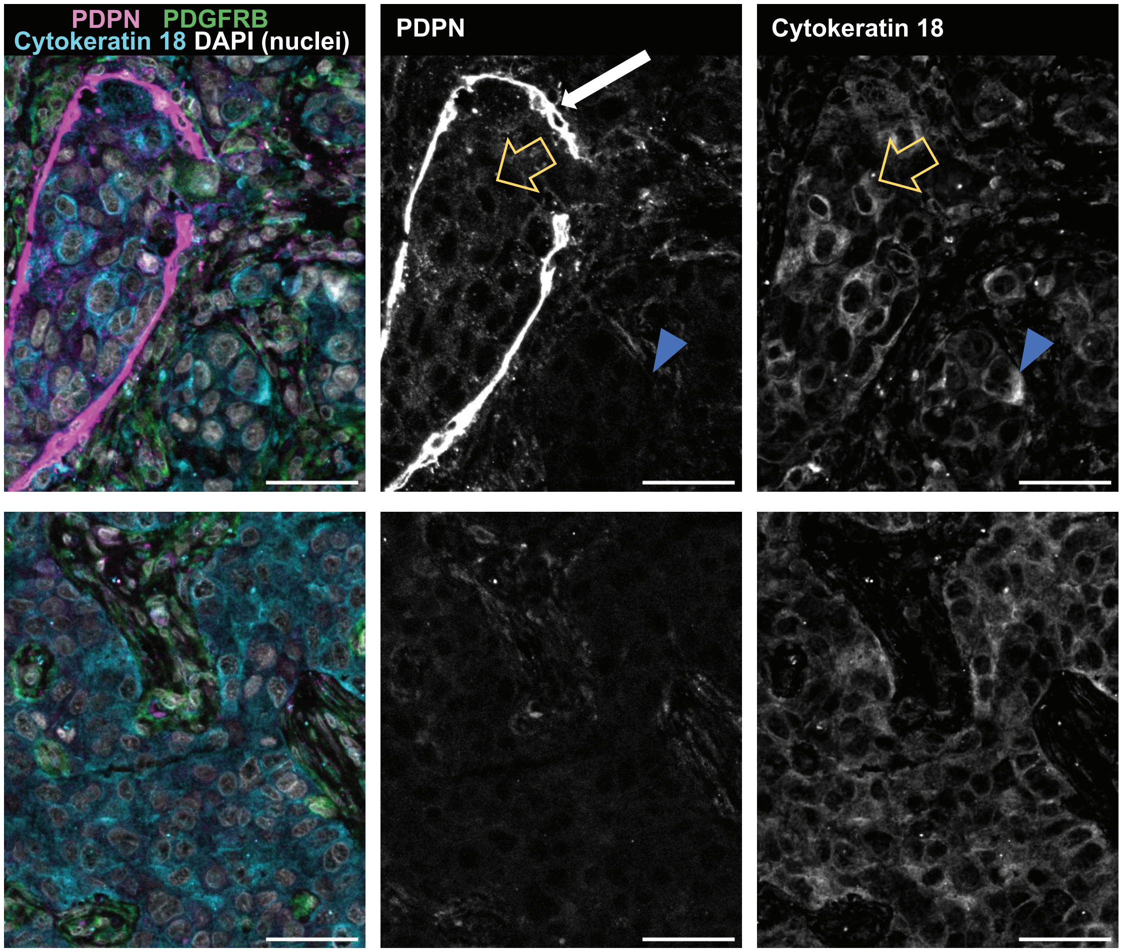

B

Tumor subtype	Cytokeratin Heterogeneity	PDPN expression
TNBC A	++++	++++
TNBC B	++	-
TNBC C	+	-
TNBC D	+	-
TNBC E	+++	+++
TNBC F	++	+
IDC A	+	-
IDC B	+	-
IDC C	+++	+
IDC D	+	-
IDC E	+	-
IDC F	+	-

Supplementary Fig. 11: Detection of PDPN in human lymph node (LN) metastatic samples. A) Representative immunofluorescence images showing PDPN (magenta/white), PDGFRB (green), cytokeratin 18 (C18; cyan/white), and DAPI (blue nuclei) in LN metastasis sections. The left column displays merged PDPN/PDGFRB/C18/DAPI images; middle and right columns show PDPN and C18 single-channel views, respectively. Scale bar: 50 µm. Upper panel: PDPN tumour cell clusters are indicated by open yellow arrows; tumour regions lacking PDPN expression are marked with blue arrowheads. Lower panel: Example from an additional patient showing absence of PDPN tumour cell clusters. B) Summary table of tumour subtype, C18 heterogeneity, and PDPN expression across analysed cases. Scoring is set relative to the TNBC 1 with the highest expression of PDPN and variable expression of C18.
